# Lifestyle after Colorectal Cancer Diagnosis in Relation to Survival and Recurrence: A Review of the Literature

**DOI:** 10.1007/s11888-017-0386-1

**Published:** 2017-09-14

**Authors:** Moniek van Zutphen, Ellen Kampman, Edward L. Giovannucci, Fränzel J. B. van Duijnhoven

**Affiliations:** 1 Division of Human Nutrition, Wageningen University and Research, P.O. Box 17, 6700 AA Wageningen, the Netherlands; 2 Department of Nutrition, Department of Epidemiology, Harvard T.H. Chan School of Public Health, 665 Huntington Avenue, Bldg. 2, Room 371, Boston, MA 02115 USA

**Keywords:** Colorectal cancer, Survival, Lifestyle, Diet, Alcohol, Physical activity, Sedentary behavior, Smoking, Body composition, Body mass index

## Abstract

**Purpose of Review:**

This review summarizes the evidence regarding diet, physical activity, smoking, and body composition after colorectal cancer (CRC) diagnosis in relation to all-cause and CRC-specific mortality and disease recurrence and gives suggestions for future research directions.

**Recent Findings:**

Overall, this review suggests that some, albeit not all, of the well-known modifiable risk factors for cancer incidence might also be associated with CRC survival. CRC prognosis appears to be worse with increased physical inactivity, smoking, or being underweight after CRC diagnosis. Emerging evidence suggests that diets associated with a positive energy balance, e.g., high consumption of sugar-sweetened beverages, may negatively impact survival in CRC survivors. In contrast, there is currently little evidence to support the recommendation to limit red and processed meat or alcohol intake after CRC diagnosis. Whether being overweight and obese after CRC diagnosis improves or worsens CRC prognosis remains controversial and may depend on the measure used to assess body fatness.

**Summary:**

Further research on post-diagnosis lifestyle patterns is needed to understand the multifactorial influence on CRC prognosis. Disease recurrence and the development of comorbidities should be included as key outcomes in future studies and lifestyle should preferably be repeatedly measured.

## Introduction

Diet, physical activity, smoking, alcohol, and body weight are associated with risk (incidence) of colorectal cancer (CRC) [1, 2]. In contrast, far fewer studies have examined the influence of these lifestyle factors on survival after CRC diagnosis. Currently, cancer survivors are advised to follow the recommendations formulated for cancer prevention [3]. However, it is currently unclear if making lifestyle changes after diagnosis would impact disease progression and survival.

Emerging evidence shows that lifestyle, including diet, after CRC diagnosis might affect all-cause and CRC-specific mortality risk. Several recent reviews and meta-analyses on observational studies summarized the available evidence on specific aspects of lifestyle, such as diet [4••, 5, 6], physical activity [4••, 5, 7–10, 11••, 12], smoking [13••, 14], and body composition [5, 10, 15, 16, 17••, 18–22], in relation to CRC outcomes. However, none of these reviews included all the aforementioned lifestyle factors in one review. Furthermore, results might differ due to the timing of lifestyle assessment (e.g., pre-diagnosis vs. post-diagnosis) [8, 10, 15] and characteristics of the included study population [15].

**Fig. 1:**
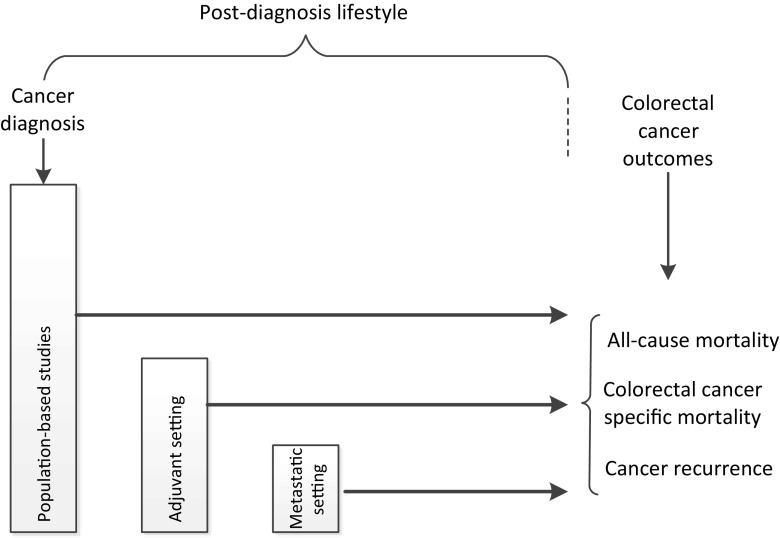
Schematic diagram of identification of three study categories based on the characteristics of the included study population. Based on the study population, studies were categorized into (1) population-based studies including all incident colorectal cancer cases, (2) studies in the adjuvant setting limited to survivors treated with adjuvant therapy, and (3) studies in the metastatic setting limited to metastatic patients. In each study category, we identified studies with lifestyle information available at or after colorectal cancer diagnosis. Studies with lifestyle information limited to the period before colorectal cancer diagnosis, either collected prospectively before diagnosis or retrospectively after diagnosis, were not taken into account

To better understand the association between lifestyle and CRC outcomes, we summarized the evidence regarding diet, physical activity, smoking, and body composition after CRC diagnosis across different groups of cancer survivors. Moreover, we also included observational studies, not included in previous reviews [23–38, 39••]. We identified three study design categories based on the selection of the included study population: (1) population-based studies including all incident CRC cases, (2) studies in the adjuvant setting limited to survivors treated with adjuvant therapy, and (3) studies in the metastatic setting limited to patients with metastatic disease (Fig. 1). We chose to focus on post-diagnosis lifestyle factors, because this is the period during which CRC survivors could be counseled to alter their behavior. Therefore, we only included studies that examined the association between lifestyle at or after CRC diagnosis and all-cause mortality, CRC-specific mortality, or cancer recurrence. Additionally, we summarized the evidence regarding changes in lifestyle, i.e., from pre- to post-diagnosis or changes made after diagnosis, among CRC survivors and survival outcomes from either observational or intervention studies. We did not include papers that examined lifestyle and CRC survival separately by molecular subtypes. These publications will be reviewed in future issue of this journal. Finally, we conclude with suggestions for future research directions.

## Overview of Included Studies

We excluded all studies that did not assess lifestyle at or after CRC diagnosis (e.g., those that assessed only pre-diagnosis factors) or did not adjust for critical confounders (e.g., age, stage). Furthermore, we excluded all studies that dichotomized body mass index (BMI) when examining the association between BMI and mortality or recurrence. Dichotomized BMI is considered a crude classification of BMI by combining diverse categories of body mass and body composition. Thus, dichotomized BMI may not account for potential differential associations between sub-categories of BMI (e.g., by combining overweight and obese in one category) [15].

**Fig. 2:**
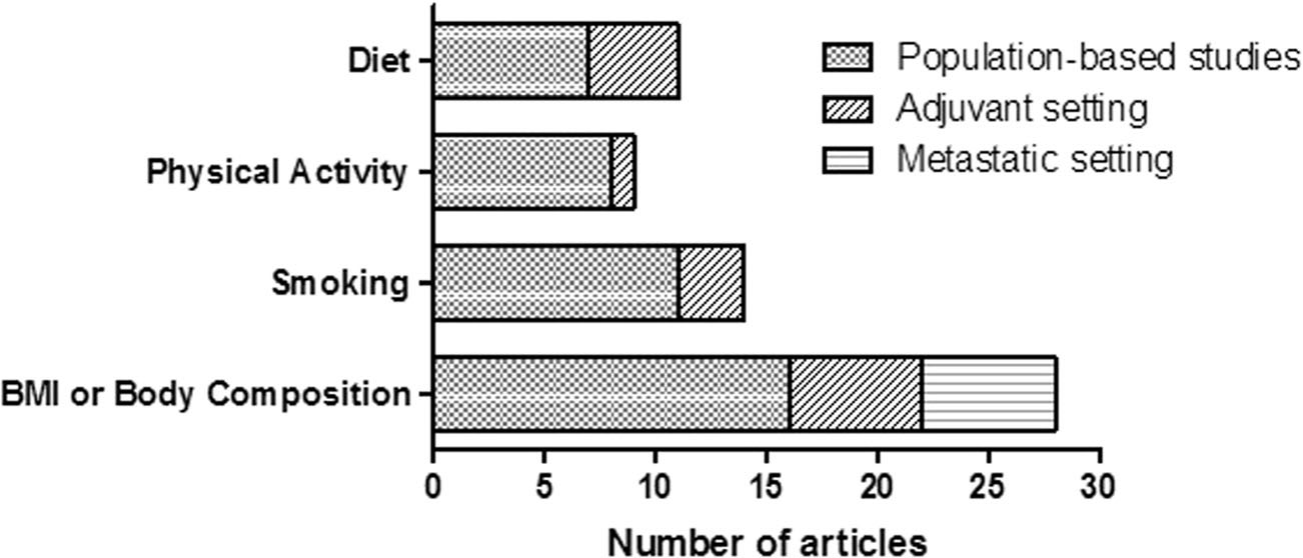
Overview of the number of included relevant articles on diet, physical activity, smoking and body mass index (BMI) or body composition at or after colorectal cancer diagnosis in relation to all-cause mortality, cancer-specific mortality, or disease recurrence by type of included study population. In total, 57 articles were included: 54 articles reported on one exposure, two articles reported on both physical activity and BMI, and one article reported on all four exposures

We included 57 relevant articles (based on 84 different observational studies) that reported on post-diagnosis diet, physical activity, smoking, or body fatness/body composition in CRC survivors in relation to all-cause mortality, CRC-specific mortality, or cancer recurrence. An overview of the number of included articles according to exposure and type of study population is shown in Fig. 2. Additionally, we included 13 relevant articles (one intervention study and 11 different observational studies) that reported on changes in lifestyle among CRC survivors in relation to survival outcomes. In total, 61 articles are discussed in more detail in this review.

## Diet after CRC Diagnosis

Five population-based studies and one study in the adjuvant setting provided results on diet and CRC outcomes in 10 publications [23–27, 40–44] (Table 1). Three US cohorts assessed post-diagnosis diet in population-based cohorts with > 1000 CRC patients: Nurses’ Health Study I (NHS) [23, 44], Health Professional Follow-Up Study (HPFS) [44], and Cancer Prevention Study (CPS) II Nutrition Cohort [27, 40, 41]. All three cohorts consist of participants diagnosed with CRC during follow-up and have updated dietary assessment after diagnosis. Usually, questionnaires that were completed after treatment was finished were utilized in the analyses. In contrast, two non-US cohorts (the German cohort PopGen [24] and BioBank Japan [26]) recruited > 1000 CRC patients after CRC diagnosis. The study in the adjuvant setting, Cancer and Leukemia Group B (CALGB) 89,803 Diet and Lifestyle Companion study [25, 42, 43], was embedded in a randomized trial of adjuvant chemotherapy among ~ 1000 patients with stage III colon cancer. Additionally, three articles, two from the CPS II Nutrition Cohort [27, 40] and one report on a small randomized dietary intervention trial reported on dietary changes among CRC survivors in relation to mortality [27, 40, 78].

**Table 1:** Cohort studies among individuals with colon or rectal cancer examining lifestyle factors after diagnosis in relation to all-cause mortality, colorectal cancer-specific mortality, or recurrence

First author, year, name of cohort, country	Study population	Time of post-diagnosis exposure assessment	Outcomes assessed	Year of CRC diagnosis and follow-up	Lifestyle factor	All-cause mortality HR (95% CI)	Colorectal cancer-specific mortality HR (95% CI)	Covariates
Dietary patterns—population-based studies								
Fung, 2014, Nurses’ Health Study I, USA [23]	*n* = 1201 W only CRC Stage I–III	≥ 6 months after CRC diagnosis (mean 21.0 months)	All-cause mortality (*n* = 435); CRC-specific mortality (*n* = 162)	Diagnosis 1986–2008; Median FU 11.2 years	Western dietary pattern Q1 Q2 Q3 Q4 Q5 *P* trend Prudent dietary pattern Q1 Q2 Q3 Q4 Q5 *P* trend Alternate Healthy Eating Index (AHEI) Q1 Q2 Q3 Q4 Q5 *P* trend Alternate Mediterranean Diet (aMED) score Q1 Q2 Q3 Q4 Q5 *P* trend Dietary Approaches to Stop Hypertension (DASH) score Q1 Q2 Q3 Q4 Q5 *P* trend	1.0 1.15 (0.83–1.58) 1.02 (0.72–1.43) 1.37 (0.97–1.94) 1.32 (0.89–1.97) 0.23 1.0 0.84 (0.62–1.13) 0.91 (0.67–1.25) 1.02 (0.73–1.42) 0.93 (0.65–1.34) 0.80 1.0 0.84 (0.63–1.10) 0.71 (0.53–0.94) 0.71 (0.52–0.96) 0.71 (0.52–0.98) 0.01 1.0 1.14 (0.85–1.52) 1.01 (0.75–1.37) 0.92 (0.66–1.27) 0.87 (0.63–1.21) 0.31 1.0 0.92 (0.68–1.24) 0.96 (0.69–1.32) 0.87 (0.65–1.18) 0.98 (0.71–1.35) 0.66	1.0 1.48 (0.87–2.54) 1.00 (0.55–1.83) 1.50 (0.84–2.70) 1.66 (0.85–3.23) 0.09 1.0 0.67 (0.40–1.12) 0.62 (0.37–1.05) 0.91 (0.53–1.55) 0.67 (0.37–1.22) 0.16 1.0 0.69 (0.42–1.12) 0.73 (0.45–1.17) 0.76 (0.47–1.23) 0.72 (0.43–1.21) 0.07 1.0 1.18 (0.73–1.91) 0.96 (0.58–1.56) 0.73 (0.42–1.20) 0.84 (0.50–1.42) 0.19 1.0 0.84 (0.52–1.34) 0.70 (0.41–1.22) 0.72 (0.43–1.20) 0.87 (0.52–1.45) 0.35	Age, PA, BMI, weight change, tumor grade, chemotherapy, smoking, energy intake, tumor site, stage, date of CRC diagnosis
Ratjen, 2017, PopGen, Germany [24]	*n* = 1404 M and W CRC Stage I–IV	6 years after diagnosis (median)	All-cause mortality (*n* = 204)	Diagnosis 1993–2005; Median FU 7 years	Modified Mediterranean Diet Score Q1 Q2 Q3 Q4 *P* trend Per 1-point increment Healthy Nordic Food Index Q1 Q2 Q3 Q4 *P* trend Per 1-point increment	1.0 0.92 (0.64–1.34) 0.85 (0.59–1.23) 0.48 (0.32–0.74) 0.001 0.88 (0.81–0.96) 1.0 0.87 (0.59–1.27) 0.77 (0.49–1.22) 0.63 (0.39–1.04) 0.06 0.90 (0.82–0.99)		Sex, age, BMI, PA, survival time from CRC diagnosis until diet assessment, tumor site, metastases, other cancer, chemotherapy, smoking, total energy intake, and time-varying age, BMI, and metastases
Dietary patterns—studies in the adjuvant setting								
Meyerhardt, 2007, CALGB 89803, USA [42]	*n* = 1009 M and W Colon Stage III	Midway through adjuvant therapy and 6 months after completion of adjuvant therapy	All-cause mortality (*n* = 251); Recurrence-free survival (*n* = 324); Disease-free survival (*n* = 352)	Diagnosis 1999–2001 Median FU 5.3 years	Western dietary pattern Q1 Q2 Q3 Q4 Q5 *P* trend Prudent dietary pattern Q1 Q2 Q3 Q4 Q5 *P* trend	1.0 0.74 (0.48–1.17) 1.38 (0.90–2.11) 1.66 (1.04–2.65) 2.32 (1.36–3.96) < 0.001 1.0 1.18 (0.81–1.71) 0.94 (0.62–1.43) 0.72 (0.46–1.13) 1.32 (0.86–2.04) 0.54	† 1.0 0.92 (0.63–1.36) 1.42 (0.98–2.07) 1.44 (0.94–2.19) 2.85 (1.75–4.63) < 0.001 † 1.0 1.07 (0.76–1.51) 1.05 (0.74–1.51) 0.83 (0.57–1.23) 1.13 (0.77–1.67) 0.84	Sex, age, depth of invasion through bowel wall, number of positive lymph nodes, presence of clinical perforation at time of surgery, presence of bowel obstruction at time of surgery, baseline performance status, treatment group, weight change between first and second questionnaire, and time-varying body mass index, PA level, and total calories
Red and processed meats—population-based studies								
McCullough, 2013, CPS II Nutrition Cohort, USA [40]	*n* = 1186 M and W CRC Stage I–III	3 years after diagnosis (mean)	All-cause mortality (*n* = 472); CRC-mortality (*n* = 146); CVD-mortality (*n* = 110); other-mortality (*n* = 216)	Diagnosis 1992–2009 Mean FU 7.6 years (SD 3.4 years)	Red and processed meat intake Q1 Q2 Q3 Q4 *P* trend	1.0 1.17 (0.89–1.55) 1.13 (0.84–1.52) 0.94 (0.68–1.30) 0.36	1.0 1.28 (0.76–2.15) 0.93 (0.53–1.64) 1.10 (0.61–1.91) 0.91	Age, sex, stage, energy intake, weight change between 1992 pre-diagnostic and post-diagnostic questionnaires, and 1992 pre-diagnostic meat intake
Fung, 2014, Nurses’ Health Study I, USA [23]	*n* = 1201 W only CRC Stage I–III	≥ 6 months after diagnosis (mean 21.0 months)	All-cause mortality (*n* = 435); CRC-specific mortality (*n* = 162)	Diagnosis 1986–2008; Median FU 11.2 years	Red/processed meat per serving/day (secondary analyses)	1.07 (0.87–1.30)	1.22 (0.90–1.67)	Age, PA, BMI, weight change, tumor grade, chemotherapy, smoking, energy intake, tumor site, stage, date of CRC diagnosis
Sugar-sweetened beverages—population-based studies								
Fung, 2014, Nurses’ Health Study I, USA [23]	*n* = 1201 W only CRC Stage I–III	≥ 6 months after diagnosis (mean 21.0 months)	All-cause mortality (*n* = 435) CRC-specific mortality (*n* = 162)	Diagnosis 1986–2008; Median FU 11.2 years	Sugar-sweetened beverages + juices per serving/d (secondary analyses)	1.11 (1.01–1.23)	1.16 (0.99–1.35)	Age, PA, BMI, weight change, tumor grade, chemotherapy, smoking, energy intake, tumor site, stage, date of CRC diagnosis
Sugar-sweetened beverages—studies in the adjuvant setting								
Fuchs, 2014, CALGB 89803, USA [43]	*n* = 1011 M and W Colon Stage III	Midway through adjuvant therapy and 6 months after completion of adjuvant therapy	All-cause mortality (*n* = 305) Recurrence-free survival (*n* = 343); Disease-free survival (*n* = 386)	Diagnosis 1999–2001 Median FU 7.3 years	Sugar-sweetened beverages intake < 2/month 2/month to 2/week 3 to 6/week 1 to < 2/day ≥ 2/day *P* trend	1.0 0.74 (0.53–1.04) 1.07 (0.75–1.53) 0.70 (0.43–1.15) 1.41 (0.79–2.50) 0.21	† 1.0 0.98 (0.72–1.34) 1.34 (0.97–1.87) 1.07 (0.70–1.65) 1.75 (1.04–2.94) 0.04	Age, sex, depth of invasion through bowel wall, number of positive lymph nodes, baseline performance status, treatment group, and the following time-varying covariates total energy intake, BMI, PA level, Western dietary pattern, prudent dietary pattern, and glycemic load
Alcohol—population-based studies								
Fung, 2014, Nurses’ Health Study, USA [23]	*n* = 1201 W only Colon and rectum Stage I–III	≥ 6 months after diagnosis (mean 21.0 months)	All-cause mortality (*n* = 435); CRC-specific mortality (*n* = 162)	Diagnosi: 1986–2008; Median FU 11.2 years	No alcohol intake 5–15 g/day > 15 g/day (secondary analyses)	1.30 (1.05–1.61) 1.0 1.22 (0.85–1.76)	1.32 (0.93–1.87) 1.0 0.97 (0.50–1.87)	Age, PA, BMI, weight change, tumor grade, chemotherapy, smoking, energy intake, tumor site, stage, date of CRC diagnosis
Lochhead, 2015, Nurses’ Health Study I + Health Professional Follow-Up Study, USA [44]	*n* = 1550 M and W CRC Stage I–III	≥ 1 year but ≤ 4 years after CRC diagnosis (median 29.5 months)	All-cause mortality (*n* = 641); CRC-specific mortality (*n* = 176)	Diagnosis up to 2006; Median FU 14.9 years	Alcohol intake g/day 0 0.1–14.9 ≥ 15 *P* trend	1.0 0.83 (0.70–0.99) 0.91 (0.72–1.16) 0.41	1.0 0.51 (0.34–0.76) 0.53 (0.28–0.98) 0.33	Pre-diagnostic alcohol consumption, age, year of diagnosis, BMI, family history of CRC, aspirin use, multivitamin use, smoking, PA, folate, vitamin B_12_, methionine, and vitamin B_6_ intake, tumor site, tumor differentiation, time from diagnosis to questionnaire return, and stage- and sex-stratified
Yang, 2017, CPS II Nutrition Cohort, USA [27]	*n* = 1599 M and W CRC Stage I–III	1.9 years after CRC diagnosis (mean)	All-cause mortality (*n* = 732); CRC-specific mortality (*n* = 235); CVD-mortality (*n* = 172); other mortality (*n* = 325)	Diagnosis: 1992–2011; Mean FU: 8.2 years (SD 4.7 years)	Alcohol drinking Never Former-former Current-former Current < 2 drinks/day Current ≥ 2 drinks/day	1.0 1.09 (0.81–1.48) 1.21 (0.92–1.60) 0.94 (0.77–1.16) 0.92 (0.66–1.26)	1.0 1.28 (0.73–2.23) 1.81 (1.13–2.91) 1.27 (0.87–1.86) 1.44 (0.80–2.60)	Age, six, tumor stage, smoking status, BMI, PA, education, and pre-existing diseases in 1982/1992
Tamakoshi, 2017, BioBank Japan, Japan [26]	*n* = 1598 M and W CRC Stage I–IV	Within 90 days after CRC diagnosis	All-cause mortality (*n* = 521)	Diagnosis 2003–2008; Median FU 7.4 years	Never drinker Ex drinker 0–15 g/day 15–30 g/day ≥30 g/day	1.0 1.26 (0.98–1.63) 0.73 (0.56–0.97) 0.79 (0.57–1.11) 0.73 (0.56–0.96)		Stratified by sex and institutions and adjusted for age and entry year
Other food groups and nutrients—population-based studies								
Yang, 2014, CPS II Nutrition cohort, USA [41]	*n* = 1111 M and W CRC Stage I–III	2.6 years after CRC diagnosis (mean)	All-cause mortality (*n* = 429); CRC-specific mortality (*n* = 143)	Diagnosis 1992–2009; Mean FU 7.6 years (SD 3.4)	Total dairy intake Q1 Q2 Q3 Q4 *P* trend Milk intake Q1 Q2 Q3 Q4 *P* trend Total calcium intake Q1 Q2 Q3 Q4 *P* trend Dietary calcium intake Q1 Q2 Q3 Q4 *P* trend Supplemental calcium intake C1 C2 C3 *P* trend Total vitamin D Q1 Q2 Q3 Q4 *P* trend Dietary vitamin D intake Q1 Q2 Q3 Q4 *P* trend	1.0 0.91 (0.69–1.21) 0.73 (0.54–0.98) 0.75 (0.56–1.01) 0.05 1.0 0.85 (0.64–1.13) 0.76 (0.52–1.12) 0.72 (0.55–0.94) 0.02 1.0 0.89 (0.67–1.18) 0.72 (0.53–0.98) 0.72 (0.53–0.98) 0.02 1.0 0.84 (0.63–1.11) 0.69 (0.51–0.93) 0.86 (0.65–1.14) 0.21 1.0 0.95 (0.72–1.27) 0.98 (0.73–1.31) 0.55 1.0 0.81 (0.59–1.10) 0.97 (0.67–1.40) 0.88 (0.57–1.35) 0.35 1.0 0.99 (0.75–1.31) 0.95 (0.71–1.27) 0.90 (0.67–1.21) 0.33	1.0 0.73 (0.44–1.23) 0.92 (0.56–1.52) 0.73 (0.44–1.23) 0.32 1.0 0.90 (0.54–1.49) 0.85 (0.44–1.67) 0.93 (0.59–1.49) 0.81 1.0 1.15 (0.71–1.86) 0.81 (0.48–1.38) 0.59 (0.33–1.05) 0.01 1.0 0.85 (0.51–1.41) 0.98 (0.59–1.62) 1.00 (0.61–1.63) 0.83 1.0 1.04 (0.65–1.69) 0.65 (0.38–1.11) 0.13 1.0 0.99 (0.59–1.66) 1.31 (0.66–2.58) 1.74 (0.80–3.77) 0.52 1.0 0.78 (0.46–1.32) 1.11 (0.67–1.85) 1.28 (0.77–2.10) 0.19	Age, sex, stage, energy intake, post-diagnostic energy intake, and total folate intakes
Fung, 2014, Nurses’ Health Study, USA [23]	*n* = 1201 W only CRC Stage I–III	≥ 6 months after diagnosis (mean 21.0 months)	Overall mortality (*n* = 435); CRC-specific mortality (*n* = 162)	Diagnosis: 1986–2008; Median FU: 11.2 years	Per serving/day Whole fruits Vegetables Nuts Whole grains (all secondary analyses)	1.08 (0.98–1.20) (0.94–1.06) 0.98 (0.82–1.17) 0.98 (0.95–1.01)	1.03 (0.87–1.21) 0.94 (0.84–1.04) 0.69 (0.49–0.97) 0.97 (0.93–1.02)	Age, PA, BMI, weight change, tumor grade, chemotherapy, smoking, energy intake, tumor site, stage, date of CRC diagnosis
Lochhead, 2015, Nurses’ Health Study I + Health Professional Follow-Up Study, USA [44]	*n* = 1550 M and W CRC Stage I–III	≥ 1 year but ≤ 4 year after CRC diagnosis (median 29.5 months)	All-cause mortality (*n* = 641); CRC-specific mortality (*n* = 176)	Diagnosis up to 2006; Median FU 14.9 years	Folate intake Q1 Q2 Q3 Q4 Q5 *P* trend Vitamin B_6_ intake Q1 Q2 Q3 Q4 Q5 *P* trend Vitamin B_12_ intake Q1 Q2 Q3 Q4 Q5 *P* trend Methionine Q1 Q2 Q3 Q4 Q5 *P* trend	1.0 1.03 (0.81–1.31) 1.17 (0.92–1.49) 0.86 (0.66–1.13) 0.87 (0.65–1.16) 0.13 1.0 0.87 (0.69–1.11) 0.80 (0.62–1.03) 0.94 (0.73–1.22) 0.78 (0.59–1.03) 0.18 1.0 1.19 (0.93–1.52) 0.96 (0.74–1.23) 0.94 (0.72–1.22) 1.11 (0.82–1.50) 0.71 1.0 0.82 (0.63–1.05) 0.92 (0.71–1.19) 1.02 (0.79–1.31) 1.17 (0.92–1.49) 0.053	1.0 1.17 (0.74–1.88) 1.63 (1.04–2.56) 0.76 (0.43–1.35) 1.04 (0.60–1.82) 0.21 1.0 0.95 (0.59–1.51) 1.08 (0.67–1.74) 0.94 (0.57–1.55) 0.93 (0.58–1.49) 0.66 1.0 1.23 (0.77–1.95) 0.70 (0.43–1.14) 0.88 (0.55–1.42) 1.04 (0.62–1.74) 0.99 1.0 0.57 (0.34–0.95) 0.82 (0.51–1.32) 0.79 (0.50–1.27) 0.90 (0.57–1.41) 0.91	Alcohol consumption, age, year of diagnosis, BMI, family history of CRC, aspirin use, multivitamin use, smoking, PA, folate, vitamin B_12_, methionine, and vitamin B_6_ intake, tumor site, tumor differentiation, time from diagnosis to questionnaire return, and stage- and sex-stratified
Tamakoshi, 2017, BioBank Japan, Japan [26]	*n* = 1598 M and W CRC Stage I–IV	Within 90 days after CRC diagnosis	All-cause mortality (*n* = 521)	Diagnosis 2003–2008 Median FU 7.4 years	Green leafy vegetable consumption Almost everyday 3–4 days/week 1–2 days/week Almost never Meat consumption Almost everyday 3–4 days/week 1–2 days/week Almost never	1.0 1.27 (0.99–1.62) 1.61 (1.18–2.20) 1.87 (1.22–2.88) 1.0 1.04 (0.76–1.41) 1.06 (0.78–1.43) 1.21 (0.85–1.71)		Stratified by sex and institutions and adjusted for age and entry year
Other food groups and nutrients—studies in the adjuvant setting								
Meyerhardt, 2012, CALGB 89803, USA [45]	*n* = 1011 M and W Colon Stage III	Midway through adjuvant therapy and 6 months after completion of adjuvant therapy	All-cause mortality (*n* = 305); Recurrence-free survival (*n* = 343); Disease-free survival (*n* = 386)	Diagnosis 1999–2001; Median FU 7.3 years	Glycemic load Q1 Q2 Q3 Q4 Q5 *P* trend Glycemic index Q1 Q2 Q3 Q4 Q5 *P* trend Fructose Q1 Q2 Q3 Q4 Q5 *P* trend Carbohydrate intake Q1 Q2 Q3 Q4 Q5 *P* trend	1.0 0.83 (0.55–1.23) 1.05 (0.72–1.54) 1.50 (1.04–2.17) 1.74 (1.20–2.51) < 0.001 1.0 0.94 (0.64–1.37) 1.22 (0.84–1.77) 1.09 (0.74–1.61) 1.23 (0.83–1.82) 0.22 1.0 0.82 (0.57–1.18) 0.74 (0.51–1.08) 0.92 (0.64–1.32) 1.11 (0.79–1.58) 0.40 1.0 1.00 (0.68–1.49) 1.11 (0.76–1.63) 1.60 (1.11–2.32) 1.80 (1.25–2.60) < 0.001	† 1.0 1.01 (0.70–1.47) 1.07 (0.74–1.56) 1.70 (1.18–2.40) 1.97 (1.39–2.79) < 0.001 1.0 0.99 (0.69–1.43) 1.21 (0.85–1.73) 1.21 (0.84–1.73) 1.24 (0.85–1.81) 0.14 1.0 0.82 (0.58–1.17) 0.95 (0.67–1.33) 1.04 (0.74–1.47) 1.43 (1.04–1.98) 0.01 1.0 1.07 (0.73–1.56) 1.20 (0.83–1.73) 1.76 (1.24–2.50) 2.06 (1.45–2.91) < 0.001	Sex, age, depth of invasion through bowel wall, number of positive lymph nodes, baseline performance status, treatment group, time-varying BMI, time-varying PA, time-varying cereal fiber, and time-varying dietary pattern
Guercio, 2015, CALGB 89803, USA [25]	*n* = 953 M and W Colon Stage III	Midway through adjuvant therapy and 6 months after completion of adjuvant therapy	All-cause mortality (*n* = 324); Recurrence-free survival (*n* = 329); Disease-free survival (*n* = 365)	Diagnosis 1999–2001 Median FU 7.3 years	Total coffee cups/day 0 < 1 1 2–3 ≥ 4 *P* trend Non-herbal tea cups/day 0 < 1 1 2–3 ≥ 4 *P* trend	1.0 0.97 (0.66–1.44) 0.97 (0.66–1.42) 0.69 (0.47–1.01) 0.66 (0.37–1.18) 0.01 1.0 1.08 (0.81–1.44) 0.87 (0.58–1.30) 0.95 (0.60–1.50) 0.82 (0.40–1.67) 0.36	† 1.0 0.98 (0.68–1.43) 0.97 (0.66–1.42) 0.80 (0.56–1.14) 0.71 (0.41–1.23) 0.07 † 1.0 1.09 (0.83–1.43) 0.89 (0.61–1.30) 1.03 (0.67–1.57) 0.86 (0.44–1.68) 0.51	Age, sex, depth of invasion through bowel wall, number of positive lymph nodes, baseline performance status, treatment group, smoking history, multivitamin, and the following time-varying covariates total energy intake, alcohol consumption, BMI, PA level, Wester dietary pattern, prudent dietary pattern, sugar-sweetened beverage intake and dietary glycemic load
Physical activity—population-based studies								
Meyerhardt, 2006, Nurses’ Health Study I, USA [46]	*n* = 554 W only CRC Stage I–III	≥ 1 year but ≤ 4 years after CRC diagnosis (median 22 months)	All-cause mortality (*n* = 121); CRC-specific mortality (*n* = 72)	Diagnosis 1986–2002; Median FU 9.6 years	Total MET-h activity/week < 3 3–8.9 9–17.9 ≥ 18 *P* trend	1.0 0.77 (0.48–1.23) 0.50 (0.28–0.90) 0.43 (0.25–0.74) 0.003	1.0 0.92 (0.50–1.69) 0.57 (0.27–1.20) 0.39 (0.18–0.82) 0.008	Age, year of diagnosis, BMI, stage, tumor grade, tumor site, chemotherapy, time from diagnosis to PA measurement, change in BMI before and after diagnosis, and smoking
Meyerhardt, 2009, Health Professional Follow-Up Study, USA [47]	*n* = 661 M only CRC Stage I–III	≥ 6 months but ≤ 4 years after CRC diagnosis (median 15 months)	All-cause mortality (*n* = 258); CRC-specific mortality (*n* = 88)	Diagnosis 1986–2004; Median FU 8.6 years	Total MET-h activity/week ≤ 3 3.1–9 9.1–18 18.1–27 ≥ 27 *P* trend	1.0 1.00 (0.68–1.48) 1.12 (0.74–1.70) 0.74 (0.46–1.20) 0.59 (0.41–0.86) < 0.001	1.0 1.06 (0.55–2.08) 1.30 (0.65–2.59) 0.76 (0.33–1.77) 0.47 (0.24–0.92) 0.002	Age, stage, tumor grade, tumor site, diagnosis year, BMI at diagnosis, time from diagnosis to PA measurement, change in BMI before and after diagnosis, and smoking
Baade, 2011, Queensland, Australia [48]	*n* = 1825 M and W CRC Stage I–III	5 months after CRC diagnosis	All-cause mortality (*n* = 462); CRC-specific mortality (*n* = 345)	Diagnosis 2003–2004; Median FU 4.9 years (range 4.0–6.0)	PA min/wk. 0 1–149 ≥ 150 *P* trend	1.0 0.72 (0.57–0.91) 0.75 (0.60–0.94) 0.007	1.0 0.90 (0.69–1.17) 0.88 (0.68–1.15) 0.585	NR
Kuiper, 2012, Women’s Health Initiative, USA [49]	*n* = 606 W only CRC Stage I–III	1.5 year after CRC diagnosis (median)	All-cause mortality (*n* = 108); CRC-specific mortality (*n* = 51)	Diagnosis ≥ 1993; Median FU 11.9 years (IQR 10.9–12.9)	Total MET-h activity /week 0 > 0–2.9 3.0–8.9 9.0–17.9 ≥ 18 *P* trend	1.0 0.71 (0.40–1.30) 0.42 (0.23–0.77) 0.57 (0.31–1.07) 0.41 (0.21–0.81) 0.005	1.0 0.49 (0.21–1.14) 0.30 (0.12–0.73) 0.53 (0.22–1.25) 0.29 (0.11–0.77) 0.02	Age, study arm, stage, ethnicity, education, alcohol, smoking, and hormone therapy use, pre-diagnostic BMI, time between baseline measurement and diagnosis
Campbell, 2013, CPS II Nutrition Cohort, USA [50]	*n* = 1800 M and W CRC Stage I–III	1.4 years after CRC diagnosis (median)	All-cause mortality (*n* = 588); CRC-specific mortality (*n* = 226); CVD-mortality (*n* = 127); Mortality from other causes (*n* = 235)	Diagnosis 1994–2007; Mean FU 6.8 years	Total MET-h activity/week < 3.5 3.5–8.74 ≥ 8.75	1.0 0.78 (0.60–1.00) 0.58 (0.47–0.71)	1.0 1.00 (0.64–1.56) 0. 87 (0.61–1.24)	Age, sex, smoking, BMI, red meat intake, stage, leisure time spent sitting, and education
Arem, 2015, National Institutes of Health-AARP, USA [51]	*n* = 1759 M and W CRC Stage I–III	4.2 years after CRC diagnosis (median)	All-cause mortality (*n* = 412); CRC-specific mortality (*n* = 128); CVD-specific mortality (*n* = 82)	Diagnosis 1996–2006; Median FU 7.1 years	PA h/wk. 0 < 1 1–3.9 4–6.9 ≥ 7 *P* trend	1.0 1.00 (0.72–1.39) 0.88 (0.65–1.19) 0.66 (0.46–0.94) 0.69 (0.49–0.98) 0.006	1.0 0.98 (0.53–1.81) 0.96 (0.57–1.62) 0.69 (0.36–1.29) 0.53 (0.27–1.03) 0.041	Sex, tumor site, tumor grade, stage, surgery, radiation, chemotherapy, time watching TV, smoking, BMI, self-reported health status, pre- and post-diagnosis PA (age is time metric in model)
Tamakoshi, 2017, BioBank Japan, Japan [26]	*n* = 1598 M and W CRC Stage I–IV	Within 90 days after diagnosis	All-cause mortality (*n* = 521)	Diagnosis 2003–2008 Median FU 7.4 years	Physical exercise ≥ 3 times/week 1–2 times/week No habit	1.0 0.60 (0.33–1.08) 1.33 (1.05–1.68)		Stratified by sex and institutions and adjusted for age and entry year
Physical activity—studies in the adjuvant setting								
Meyerhardt, 2006, CALGB 89803, USA [52]	*n* = 832 M and W Colon Stage III	7.1 months after completion of adjuvant treatment (median)	All-cause mortality (*n* = 84); Recurrence-free survival (*n* = 159); Disease-free survival (*n* = 172)	Inclusion 1999–2001; Median FU 2.7 years	Total MET-h activity/week < 3 3–8.9 9–17.9 18–26.9 ≥ 27 *P* trend	1.0 0.85 (0.49–1.49) 0.71 (0.36–1.41) 0.71 (0.32–1.59) 0.37 (0.16–0.82) 0.01	† 1.0 0.86 (0.57–1.30) 0.89 (0.55–1.42) 0.51 (0.26–1.01) 0.60 (0.36–1.01) 0.03	Age, sex, depth of invasion through bowel wall, no. of positive lymph nodes, clinical perforation at time of surgery, baseline CEA, tumor, baseline performance status, treatment arm, weight change between first and second questionnaire, BMI at time of second questionnaire, time between study entry, and completion of second questionnaire
Sedentary behavior—population-based studies								
Campbell, 2013, CPS II Nutrition Cohort, USA [50]	*n* = 1656 M and W CRC Stage I–III	1.9 years after CRC diagnosis (median)	All-cause mortality (*n* = 477); CRC-specific mortality (*n* = 169); CVD-mortality (*n* = 110); Mortality from other causes (*n* = 198)	Diagnosis 1994–2007; Mean FU 6.8 years	Leisure time spent sitting < 3 h/day 3–< 6 ≥ 6 h/day	1.0 1.13 (0.91–1.40) 1.27 (0.99–1.64)	1.0 1.23 (0.84–1.78) 1.62 (1.07–2.44)	Age, sex, smoking, BMI, red meat intake, stage, PA, and education
Arem, 2015, National Institutes of Health-AARP, USA [51]	*n* = 1759 M and W CRC Stage I–III	4.2 years after diagnosis (median)	All-cause mortality (*n* = 412); CRC-specific mortality (*n* = 128); CVD-specific mortality (*n* = 82)	Diagnosis 1996–2006; Median FU 7.1 years	TV viewing 0–2 h/day 3–4 h/day ≥ 5 h/day *P* trend	1.0 0.98 (0.75–1.27) 1.25 (0.93–1.67) 0.126	1.0 0.90 (0.56–1.46) 1.45 (0.85–2.47) 0.156	Age as time metric. Sex, tumor site, tumor grade, stage, chemotherapy, PA, smoking, BMI, self-reported health, and pre-diagnosis TV viewing
Cao, 2015, Health Professional Follow-Up Study, USA [53]	*n* = 714 M only CRC Stage I–III	≥ 6 months but ≤ 3 years after CRC diagnosis	All-cause mortality (*n* = 325); CRC-specific mortality (*n* = 72); Mortality from other causes (*n* = 253)	Diagnosis 1986–2010; FU until end 2011	Sitting watching TV 0–6 h/week 7–13 h/week 14–20 h/week ≥ 21 h/week *P* trend	1.0 0.98 (0.70–1.37) 1.01 (0.72–1.42) 1.16 (0.80–1.68) 0.66	1.0 0.62 (0.27–1.41) 0.68 (0.30–1.54) 1.45 (0.73–2.87) 0.27	Age, year of diagnosis, stage, tumor grade, tumor site, smoking, PA, BMI, AHEI, and pre-diagnosis TV viewing
Smoking—population based studies								
Jadallah, 1999, Dunedin hospital, New Zealand [54]	*n* = 241 M and W CRC Stage I–III	Hospital record	All-cause mortality (*n* = 81)	Diagnosis 1990–1992; FU 5 years	Non-smoker Smoking	1.0 2.26 (1.31–3.90)		Blood transfusion, stage
Ali, 2011, Irish National Cancer Registry, Ireland [55]	*n* = 22,335 M and W CRC Stage I–IV	Cancer registry	All-cause mortality (*n* = 11,400);	Diagnosis 1994–2005; Max FU 15 years	Former smoker Current smoker Never smoker Current smoker	1.0 1.15 (1.07–1.23) 1.0 1.20 (1.13–1.28)		Age, tumor grade, stage
Warren, 2013, Roswell Park Cancer Institute, USA [56]	*n* = 359 M and W CRC Stage I–IV	Within 1 month after CRC diagnosis	All-cause mortality (*n* = NR); CRC-specific mortality (*n* = NR):	Diagnosis 1982–1998 FU 12–27.7 years	Men Former smoker Current smoker Never smoker Current smoker Women Former smoker Current smoker Never smoker Current smoker	1.0 1.07 (0.64–1.81) 1.0 1.05 (0.62–1.78) 1.0 0.89 (0.39–2.06) 1.0 1.70 (0.87–3.31)	1.0 1.14 (0.56–2.27) 1.0 0.70 (0.36–1.36) 1.0 1.18 (0.34–4.05) 1.0 1.85 (0.85–4.02)	Disease site, sex, age, stage, race, date of diagnosis, BMI, total pack-years of smoking
Tao, 2013, Shanghai Cohort Study, China [28]	*n* = 248 M only CRC Stage NR	At diagnosis and yearly thereafter	All-cause mortality (*n* = 152)	Diagnosis 1986–2010; Mean FU 5.3 (±4.8) years	Non-smoking Smoking (time-dependent)	1.0 1.65 (1.14–2.38)		Age, education, pack-years of smoking before diagnosis, treatment, and cancer site
Amri, 2015, Massachusetts General Hospital, USA [57]	*n* = 1071 M and W CRC Stage I–IV	At pre-operative assessment	All-cause mortality (*n* = NR); CRC-specific mortality (*n* = NR); Metastatic recurrence (*n* = NR)	Diagnosis 2004–2011; FU NR	Non-smoking Current smoking *P* trend	1.0 1.44 (1.07–1.94) 0.017	1.0 1.21 (0.80–1.83) 0.36	Age, stage, BMI, comorbidities
Walter, 2015, DACHS study, Germany [58]	*n* = 3130 M and W CRC Stage I–IV	24 days after CRC diagnosis (median)	All-cause mortality (*n* = 889); CRC-specific mortality (*n* = 644); Recurrence-free survival (*n* = 828); Disease-free survival (*n* = 1024); Non-CRC related mortality (*n* = 232)	Diagnosis 2003–2010; Median FU 4.9 years (IQR 2.9–5.1)	Non-smoking < 15 cigarettes/day ≥ 15 cigarettes/day	1.0 1.10 (0.85–1.43) 0.99 (0.73–1.32)	1.0 1.08 (0.83–1.41) 1.14 (0.87–1.51)	Age, sex, BMI, stage, alcohol consumption, red meat consumption, family history of CRC, use of statins, use of NSAIDs, use of beta blockers, diabetes mellitus, history of heart failure, myocardial infarction, angina pectoris or stroke, history of nonCRC cancer; additional adjustment for age × log(time) and cancer × log(time)
Yang, 2015, CPS II Nutrition Cohort, USA [29]	*n* = 2256 M and W CRC Stage I–III	1.4 years after CRC diagnosis (mean)	All-cause mortality (*n* = 865); CRC-specific mortality (*n* = 324)	Diagnosis 1992–2009; Mean FU 7.5 years (SD 4.6 years)	Never smoking Former smoking Current smoking	1.0 1.21 (1.03–1.42) 2.22 (1.58–3.13)	1.0 0.91 (0.71–1.18) 1.92 (1.15–3.21)	Age, sex, stage, alcohol consumption, BMI, and PA
Sharp, 2017, National Cancer Registry Ireland, Ireland [14]	*n* = 18,166 M and W Colon Stage I–IV	At diagnosis	CRC-specific mortality (*n* = 7488)	Diagnosis 1994–2012; FU 5 years	Never smoker Ex-smoker Current smoker *P* trend		1.0 1.00 (0.94–1.07) 1.14 (1.07–1.22) < 0.01	Sex, marital status, deprivation category, period of diagnosis, grade, tumor site. With stage and age fitted as stratification factors
Sharp, 2017, National Cancer Registry Ireland, Ireland [30]	*n* = 10,794 M and W Rectum Stage I–IV	At diagnosis	CRC-specific mortality (*n* = 4491)	Diagnosis 1994–2012; FU 5 years	Never smoker Ex-smoker Current smoker *P* trend		1.0 1.02 (0.93–1.11) 1.15 (1.06–1.24) < 0.01	Sex, marital status, deprivation category, period of diagnosis, grade. With stage and age fitted as stratification factors
Rasouli, 2017, Kurdistan’s Cancer Registry, Iran [31]	*n* = 335 M and W CRC Stage II–III	Medical record	All-cause mortality (*n* = 164)	Diagnosis 2009–2014; Median FU 42.6 ± 2.8 months	Non-smoking Smoking	1.0 1.34 (0.92–1.95)		Age, residence, marital status, occupation, education, socioeconomic status, comorbidity, stage, tumor grade
Tamakoshi, 2017, BioBank Japan, Japan [26]	*n* = 1598 M and W CRC Stage I–IV	Within 90 days after CRC diagnosis	All-cause mortality (*n* = 521)	Diagnosis 2003–2008; Median FU 7.4 years	Never smoker Ex-smoker Current smoker	1.0 1.27 (1.02–1.59) 1.38 (1.06–1.81)		Stratified by sex and institutions and adjusted for age and entry year
Smoking—studies in the adjuvant setting								
Munro, 2006, Tayside Cancer Centre, UK [59]	*n* = 284 M and W CRC Stage NR	At the first assessment in the oncology department, usually around 4 weeks after surgery	CRC-specific mortality (*n* = 83)	Diagnosis: 1997–1999; Median FU: 56 months (range 20–83)	Non-smoker Current smoker		1.0 2.24 (1.25–4.01)	Number of positive nodes, deprivation, co-morbidity, T stage
McCleary, 2010, CALGB 89803, USA [60]	*n* = 1045 M and W Colon Stage III	4 months after surgery	All-cause mortality (*n* = 257); Recurrence-free survival (*n* = 332); Disease-free survival (*n* = 363);	Diagnosis 1999–2001; Median FU: 5.3 years	Never smoker Former smoker Current smoker	1.0 1.17 (0.87–1.57) 1.38 (0.87–2.18)	† 1.0 1.15 (0.89–1.48) 0.90 (0.58–1.41)	Age, sex, number of positive lymph nodes, extent of invasion through bowel wall, tumor differentiation, BMI, and clinical bowel obstruction at diagnosis
Phipps, 2013, North Central Cancer Treatment Group N0147, USA [61]	*n* = 1968 M and W Colon Stage III	Within 56 days after surgery	Time-to-recurrence (*n* = NR); Disease-free survival (*n* = NR)	Diagnosis 2004–2009; Median FU 3.5 years	Never smoker Former smoker Current smoker		† 1.0 1.19 (0.97–1.46) 1.47 (1.03–2.11)	Tumor site, number of involved lymph nodes, T stage, mismatch repair status, performance score, PA, BMI, alcohol consumption, age, and sex
BMI—population-based studies								
Asghari-Jafarabadi, 2009, Shahid Beheshti Medical University, Iran [62]	*n* = 1219 M and W CRC Stage I–IV	Hospital record	All-cause mortality (*n* = NR)	Diagnosis NR Mean FU 2.1 years	BMI < 18.5 18.5–24.9 25.0–29.9 ≥ 30	2.74 (1.17–6.45) 1.0 0.32 (0.14–0.73) 0.71 (0.25–2.03)		Age, alcohol history, inflammatory bowel disease, tumor grade, stage
Hines, 2009, University of Alabama at Birmingham Hospital, USA [63]	*n* = 496 M and W Colon Stage I–IV	At time of surgery	All-cause mortality (*n* = 333)	Diagnosis 1981–2002 FU until 2008	BMI < 18.5 18.5–24.9 ≥ 25	1.54 (0.96–2.45) 1.0 0.77 (0.61–0.97)		Age, ethnicity, comorbidity, stage, tumor grade, bowel obstruction
Baade, 2011, Queensland, Australia [48]	*n* = 1825 M and W CRC Stage I–III	5 months after diagnosis	All-cause mortality (*n* = 462); CRC-specific mortality (*n* = 345)	Diagnosis 2003–2004; Median FU 4.9 years (range 4.0–6.0)	BMI < 18.5 18.5–24.9 25.0–29.9 ≥ 30	2.29 (1.47–3.59) 1.0 0.75 (0.61–0.94) 0.78 (0.59–1.03)	1.74 (1.00–3.04) 1.0 0.75 (0.59–0.97) 0.70 (0.51–0.97)	NR
Campbell, 2012, CPS II Nutrition Cohort, USA [64]	*n* = 1957 M and W Colon Stage I–III	18 months after diagnosis	All-cause mortality (*n* = 815); CRC-specific mortality (*n* = 380); CVD-specific mortality (*n* = 153)	Diagnosis 1994–2007; Median FU 6.4 years (range 2 days–16.1 years)	BMI < 18.5 18.5–24.9 25.0–29.9 ≥ 30	1.30 (0.82–2.06) 1.0 0.83 (0.70–1.00) 0.93 (0.75–1.17)	0.64 (0.25–1.60) 1.0 0.87 (0.65–1.17) 1.14 (0.81–1.60)	Age, smoking, PA, red meat intake, stage
Chin, 2012, Taiwan [65]	*n* = 2135 M and W Colon Stage I–III	NR	All-cause mortality (*n* = NR); CRC-specific mortality (*n* NR); Disease-free survival (*n* NR)	Diagnosis 1995–2003; FU at least 5 years or until death	BMI < 18.5 18.5–24.9 25.0–29.9 ≥ 30	1.58 (1.23–2.05) 1.0 0.83 (0.68–1.01) 0.94 (0.74–1.18)	1.33 (0.94–1.87) 1.0 0.96 (0.76–1.22) 1.06 (0.80–1.41)	Stage, age, sex, comorbidities, CEA, hemoglobin, albumin, timing of surgery, postoperative morbidity, tumor site, histolic type, tumor grade
Kuiper, 2012, Women’s Health Initiative, USA [49]	*n* = 587 W only CRC Stage I–III	0.8 (IQR 0.4–1.7) years after diagnosis (median)	All-cause mortality (*n* = 108); CRC-specific mortality (*n* = 51)	Diagnosis ≥ 1993 Median FU 11.9 years (IQR 10.9–12.9)	BMI 18.5–25.0 25.0–30.0 ≥ 30	1.0 0.77 (0.47–1.27) 1.09 (0.65–1.83)	1.0 0.45 (0.22–0.92) 0.95 (0.49–1.85)	Age, study arm, stage, ethnicity, education, alcohol, smoking, and hormone therapy use, pre-diagnostic BMI, time between baseline measurement and diagnosis
Alipour, 2013, British Columbia Cancer Agency, Canada [66]	*n* = 913 M and W Colon Stage II–III	Recorded at initial consultation	All-cause mortality (*n* = NR); CRC-specific mortality (*n* = NR); Relapse-free survival (*n* = NR)	Diagnosis 2001–2005; Median FU 6.9 (IQR 5.2–8.5) years	BMI 18.5–25.0 25.0–30.0 ≥ 30	1.0 0.89 (0.71–1.11) 1.02 (0.78–1.33)	1.0 0.80 (0.61–1.05) 1.05 (0.77–1.42)	Age, gender, stage, number of lymph nodes retrieved, and systemic therapy
Schlesinger, 2014, PopGen, Germany [16]	*n* = 2143 M and W CRC Stage I–IV	4 years after diagnosis (mean)	All-cause mortality (*n* = 349)	Diagnosis 2002–2005; Mean FU 3.5 years	BMI < 18.5 18.5–24.9 25.0–29.9 ≥ 30 *P* trend	1.65 (0.79–3.46) 1.0 0.80 (0.62–1.02) 0.84 (0.62–1.14) 0.09		Age, sex, alcohol, smoking, tumor site, family history of CRC, metastases and other cancer
Kroenke, 2016, Kaiser Permanente Northern California, USA [32]	*n* = 3408 M and W CRC Stage I–III	At diagnosis and 15 months after diagnosis	All-cause mortality (*n* = 617); CRC-specific mortality (*n* = 411)	Diagnosis 2006–2011; Median FU 3.5 (range 0.0–7.9) years	BMI at diagnosis < 18.5 18.5–24.9 25.0–29.9 30–34.9 ≥ 35 BMI after diagnosis < 18.5 18.5–22.9 23–24.9 25.0–27.9 28–29.9 30–34.9 ≥ 35	3.01 (1.88–4.83) 1.0 0.81 (0.64–1.03) 1.03 (0.77–1.38) 1.63 (1.13–2.33) 3.38 (2.19–5.20) 1.0 0.72 (0.52–1.02) 0.56 (0.41–0.77) 0.39 (0.26–0.58) 0.51 (0.35–0.73) 0.85 (0.56–1.30)	3.35 (1.92–5.87) 1.0 0.77 (0.57–1.03) 1.06 (0.75–1.50) 1.47 (0.96–2.27) 3.21 (1.88–5.47) 1.0 0.69 (0.46–1.05) 0.50 (0.34–0.75) 0.42 (0.26–0.67) 0.56 (0.36–0.85) 0.84 (0.51–1.37)	Sociodemographics, disease severity, treatment, and pre-diagnosis BMI
Walter, 2016, DACHS, Germany [33]	*n* = 3130 M and W CRC Stage I–IV	At diagnosis	All-cause mortality (*n* = 896); CRC-specific mortality (*n* = 649); Recurrence-free survival (*n* = 828); Disease-free survival (*n* = 1024)	Diagnosis 2003–2010; Median FU 4.9 years	BMI < 20 20–24.9 25.0–29.9 ≥ 30	1.21 (0.89–1.66) 1.0 0.82 (0.70–0.95) 0.80 (0.66–0.98)	0.95 (0.65–1.41) 1.0 0.84 (0.71–1.01) 0.78 (0.62–0.99)	Age, sex, tumor site, stage, alcohol, smoking, use of statins, use of NSAIDs, use of beta-blockers, hyperlipidemia, diabetes mellitus, history of heart failure, myocardial infarction, angina pectoris or stroke, history of other cancer, age × log(time) and history of other cancer × log(time)
Tamakoshi, 2017, BioBank Japan, Japan [26]	*n* = 1598 M and W CRC Stage I–IV	Within 90 days after diagnosis	All-cause mortality (*n* = 521)	Diagnosis 2003–2008 Median FU 7.4 years	BMI < 18.5 18.5–24.9 25.0–29.9 ≥ 30	1.40 (1.12–1.76) 1.0 0.80 (0.62–1.05) 1.54 (0.86–2.76)		Stratified by sex and institutions and adjusted for age and entry year
BMI—studies in the adjuvant setting								
Meyerhardt, 2003, Intergroup Trial 0089, USA [67]	*n* = 3438 M and W Colon Stage II–III	Day 1 of chemotherapy	All-cause mortality (*n* = NR); Recurrence-free survival (*n* = NR); Disease-free survival (*n* = NR)	Diagnosis 1988–1992; Median FU 9.4 years (max 12.7)	BMI < 21 21.0–24.9 25.0–27.49 27.5–29.9 ≥ 30 *P* trend	1.15 (0.98–1.35 1.0 1.10 (0.95–1.26) 1.05 (0.90–1.24) 1.11 (0.96–1.29) 0.20	† 1.06 (0.88–1.27) 1.0 1.06 (0.88–1.27) 1.12 (0.94–1.33) 1.11 (0.94–1.30) 0.17	Age, sex, race, performance status, bowel obstruction, bowel perforation, stage, peritoneal implants, predominant macroscopic pathologic feature, completion of chemotherapy
Meyerhardt, 2004, Intergroup Trial 0114, USA [68]	*n* = 1688 M and W Rectum Stage II–III	Day 1 of chemotherapy	All-cause mortality (*n* = NR); Recurrence-free survival (*n* = NR); Disease-free survival (*n* = NR)	Diagnosis 1990–1992; Median FU 9.9 years (max 11.8)	BMI < 20 20.0–24.9 25–26.9 27–29.9 ≥ 30 *P* trend	1.43 (1.08–1.89) 1.0 0.97 (0.80–1.17) 0.95 (0.78–1.15) 1.09 (0.90–1.33) 0.5	† 1.16 (0.85–1.58) 1.0 1.01 (0.81–1.24) 0.88 (0.71–1.09) 1.08 (0.87–1.33) 0.8	Age, sex, race, performance status, bowel obstruction, extent of bowel wall invasion, number of positive lymph nodes
Sinicrope, 2013, pooled analyses ACCENT database (21 studies), USA [69]	*n* = 25,291 M and W CRC Stage II–III	At study enrolment	All-cause mortality (*n* = 7973); Time to Recurrence (*n* = 7973); Disease-free survival (*n* = 15,946);	Diagnosis NR; Median FU 7.8 years	BMI < 20 20–24.9 25.0–29.9 ≥ 30 30–34.9 ≥ 35 *P* trend	1.21 (1.11–1.32) 1.0 0.99 (0.94–1.04) 1.10 (1.04–1.17) 1.10 (1.02–1.18) 1.11 (1.00–1.23) < 0.0001	† 1.13 (1.04–1.24) 1.0 0.99 (0.94–1.04) 1.06 (1.00–1.13) 1.05 (0.98–1.20) 1.08 (0.98–1.20) 0.007	Age, stage, treatment, sex
BMI—studies in the metastatic setting								
Patel, 2015, South Australia Clinical registry for metastatic CRC, Australia [34]	*n* = 1174 M and W CRC Stage IV	At first diagnosis of metastatic CRC, prior to treatment with chemotherapy	All-cause mortality (*n* = NR)	Diagnosis ≥ 2006; Median FU 24 months	BMI < 18.5 18.5–24.9 25.0–29.9 30.0–34.9 ≥ 35	2.21 (1.53–3.19) 1.0 1.23 (1.03–1.46) 1.20 (0.94–1.51) 0.89 (0.64–1.23)		Age, sex, synchronous disease, > 1 met site, number of lines of chemotherapy and number of lines of antibody
Renfro, 2016, ARCAD database (25 studies) [70]	*n* = 21,149 M and W CRC Stage IV	Baseline BMI	All-cause mortality (*n* = NR); Progression-free survival (*n* = NR)	Diagnosis 1997–2012; Median FU 18.9 months	Continuous BMI	*P* < 0.001 with an L-shaped pattern; highest risk for patients with the lowest BMI, it decreased until a BMI of approximately 28 kg/m^2^, and remained similar for patients with higher BMI		Age, sex, performance score, cancer site, number of metastatic sites; previous chemotherapy usage; presence of liver, lung and lymph node metastases
Visceral adipose tissue—population-based studies								
Rickles, 2013, University of Rochester Medical Center, USA [71]	*n* = 219 M and W CRC Stage I–III	CT, preoperative visceral fat volume	All-cause mortality (*n* = NR); Recurrence-free survival (*n* = 34); Disease-free survival (*n* = NR)	Diagnosis 2003–2010; Max FU 96 months	Visceral fat volume Stage I < median > median Stage II < median > median Stage III < median > median	1.0 0.67 (0.18–2.59) 1.0 1.97 (0.78–5.02) 1.0 0.43 (0.17–1.07)	† Insufficient number of events † 1.0 3.76 (1.12–12.57) † 1.0 0.39 (0.16–0.99)	Major complication, intraoperative blood transfusion, laparoscopic approach, smoking history, gender, age, use of adjuvant or neoadjuvant chemotherapy, and tumor size
Black, 2017, Aberdeen Royal Infirmary, UK [35]	*n* = 339 M and W CRC Stage I–III	CT, preoperative visceral fat index	All-cause mortality (*n* = 213)	Diagnosis 2006–2014; Median FU 62 months (range 3–105)	Visceral fat index High Medium Low	1.00 (0.80–1.26)		Age, sex, stage, neoadjuvant therapy, adjuvant therapy, lymphovascular invasion, neutrophil count, subcutaneous fat index, skeletal muscle index
Caan, 2017, Kaiser Permanente Northern California, USA [39••]	*n* = 3262 M and W CRC Stage I–III	CT, within 4 months of diagnosis and before chemotherapy or radiation, visceral fat area	All-cause mortality (*n* = 788); CRC-specific mortality (*n* = 433)	Diagnosis 2006–2011; Median FU 5.8 years (range 0.0–9.9)	Body composition Normal High visceral adiposity and normal muscle High visceral adiposity and low muscle	1.0 1.22 (0.99–1.49) 1.40 (1.05–1.87)		Age, sex, race, stage, chemotherapy, radiation, tumor site, partitioned BMI, subcutaneous adiposity
Visceral adipose tissue—studies in the adjuvant setting								
Clark, 2013, Moffit Cancer Center, USA [72]	*n* = 96 M and W Rectum Stage II–III	CT, diagnostic visceral fat area to subcutaneous fat area ratio and perinephric fat thickness	All-cause mortality (*n* = NR); Disease-free survival (*n* = 26)	Diagnosis 1998–2010; max FU 7 years	Visceral fat area to subcutaneous fat area ratio < 0.4 ≥ 0.4 Perinephric fat thickness, mm	1.0 2.03 (0.57–7.20) 1.04 (0.99–1.09)		Grade and pathologic response
Lee, 2015, St. Vincent’s University Hospital, Ireland [73]	*n* = 62 M and W CRC Stage I–III	CT, preoperative visceral fat area	All-cause mortality (*n* = NR); Disease-free survival (*n* = NR)	Diagnosis 2006–2009; Median FU 62.5 months	Visceral fat area < 130 cm^2^ > 130 cm^2^	1.0 7.0 (2.0–24.6)		T stage, N stage
Visceral adipose tissue—studies in the metastatic setting								
Guiu, 2010, Georges-François Leclerc Cancer Centre, France [74]	*n* = 120 M and W CRC Stage IV	CT, pre-treatment visceral fat area	All-cause mortality (*n* = 22); Disease progression (*n* = 92)	Diagnosis 2002–2008; Mean FU 24 months	Visceral fat area, cm^2^ Bevacizumab group < 117.88 ≥ 117.58 *P* Chemotherapy group < 117.88 ≥ 117.58 *P*	1.0 2.88 0.027 1.0 NR NS		Performance status, CEA, high subcutaneous fat area
Muscle mass—population-based studies								
Miyamoto, 2015, Kumamoto University Hospital, Japan [75]	*n* = 220 M and W CRC Stage I–III	CT, preoperative skeletal muscle index	All-cause mortality (*n* = 37); Recurrence-free survival (*n* = 85)	Diagnosis 2005–2010; Median FU 41.4 months	Skeletal muscle index Q1–3 Q4	1.0 2.27 (1.15–4.49)	† 1.0 2.18 (1.20–3.94)	Sex, performance score, tumor site, histological findings, preoperative serum CEA level
Malietzis, 2016, St Mark’s Hospital, UK [38]	*n* = 805 M and W CRC Stage I–IV	CT, preoperative skeletal muscle index	All-cause mortality (*n* = 156); Disease-free survival (*n* = 101)	Diagnosis 2006–2011; Median FU 47 months (IQR 24.9–65.6)	Skeletal muscle index Normal Low	1.0 1.70 (1.25–2.31)		Age, ASA score, surgical approach, stage, tumor grade, lymphovascular invasion, adjuvant chemotherapy
Black, 2017, Aberdeen Royal Infirmary, UK [35]	*n* = 339 M and W CRC Stage I–III	CT, preoperative skeletal muscle index	All-cause mortality (*n* = 213)	Diagnosis 2006–2014; Median FU 62 months (range 3–105)	Skeletal muscle index Normal Low	1.0 0.76 (0.35–1.65)		Age, sex, stage, neoadjuvant therapy, adjuvant therapy, lymphovascular invasion, neutrophil count, subcutaneous fat index, visceral fat index
Caan, 2017, Kaiser Permanente Northern California, USA [39••]	*n* = 3262 M and W CRC Stage I–III	CT, within 4 months of diagnosis and before chemotherapy or radiation, skeletal muscle index and muscle cross-sectional area	All-cause mortality (*n* = 788); CRC-specific mortality (*n* = 433)	Diagnosis 2006–2011; Median FU 5.8 years (range 0.0–9.9)	Skeletal muscle index Normal Low Muscle, cm^2^ Low tertile 1 Middle tertile 2 High tertile 3 *P* trend	1.0 1.27 (1.09–1.48) 1.32 (1.07–1.64) 1.13 (0.93–1.37) 1.0 0.01	1.0 1.46 (1.19–1.79) 1.54 (1.16–2.05) 1.19 (0.92–1.55) 1.0 0.003	Age, sex, race, stage, chemotherapy, radiation, tumor site, partitioned BMI, total adiposity
Muscle mass—studies in the adjuvant setting								
Jung, 2015, Seoul National University Bundag Hospital, South Korea [37]	*n* = 229 M and W Colon Stage III	CT, preoperative psoas muscle cross-sectional area	All-cause mortality (*n* = 30); Disease-free survival (*n* NR)	Diagnosis 2003–2010; Median FU 61.3 months (IQR 49.7–72.0)	1 SD decrement in the psoas index	1.85 (1.10–3.13)		Age, sex, T stage, N stage, chemotherapy dose intensity, comorbidities, and BMI
Muscle mass—studies in the metastatic setting								
van Vledder, 2012, Erasmus Medical Center, the Netherlands [76]	*n* = 196 M and W CRC Stage IV	CT, perioperative skeletal muscle mass	All-cause mortality (*n* = 84); Disease-free survival (*n* NR)	Diagnosis2001–2009; Median FU 29 (1–97) months	Skeletal muscle mass Normal Low	1.0 2.69 (1.67–4.32)		No. of metastases, radiofrequency ablation, resection margin
Thoresen, 2013, St. Olav’s University Hospital/Cross Cancer Institute, Norway/Canada [77]	*n* = 71 M and W CRC Stage IV	CT, skeletal muscle mass cross-sectional area	All-cause mortality (*n* = 60)	Diagnosis 2004–2006; Median FU 15.8/20.6 months	Skeletal muscle mass Normal Low	1.0 1.74 (0.99–3.03)		Nation, age, and gender
Blauwhoff-Buskermolen, 2016, Vrije Universiteit Medical Center, the Netherlands [36]	*n* = 67 M and W CRC Stage IV	CT, skeletal muscle area	Overall mortality (*n* NR)	Diagnosis 2011–2014; Median FU 17.5 months (95% CI 13.3–21.7) for patients receiving first-line chemotherapy and 8.5 months (95% CI 4.4–12.6) for patients receiving second-line chemotherapy or beyond	Muscle mass Normal Low	1.0 1.65 (0.85–3.18)		Sex, age, lactate dehydrogenase concentration, comorbidity, metastases, chemotherapy line

*CRC* colorectal cancer, *HR* hazard ratio, *95% CI* 95% confidence interval, *SD* standard deviation, *IQR* interquartile range, *M* men, *W* women, *NR* not reported, *NS* non-significant, *Q* quintile or quartile, *C* category, *BMI* body mass index, *PA* physical activity, *MET-h* metabolic equivalent task-hour, *CT* computed tomography, *CEA* carcinoembryonic antigen, *MSI* microsatellite instability, *CALGB* Cancer and Leukemia Group B, *CPS II* Cancer Prevention Study II, *DACHS* German: Darmkrebs: Chancen der Verhutüng durch Screening, English: chances for prevention through screening; *ACCENT* Adjuvant Colon Cancer Endpoints, *ARCAD* Aide et Recherche en Cancérologie Digistive

^†^Results are for disease recurrence

In this review, we summarized the available evidence for dietary patterns, red and processed meat, sugar-sweetened beverages, alcohol consumption, other foods and beverages, and CRC survival.

### Dietary Patterns

Two observational studies, the NHS I [23] and a German cohort of CRC survivors [24], assessed post-diagnosis dietary patterns in a population-based setting [23], while CALGB 89803 [42] reported results in the adjuvant setting (Table 1). Data-driven dietary patterns were assessed within NHS I [23] and CALGB 89803 [42]. Both studies observed patterns that were given the labels a “Western” and a “Prudent” dietary pattern. The Western dietary pattern was characterized by high- and low-fat dairy, refined grains, red and processed meats, desserts, and potatoes, while the Prudent dietary pattern was characterized by high intakes of fruits, vegetables, whole grains, and poultry.

For the Western dietary pattern, both studies reported an increased all-cause mortality risk [23, 42]. However, the association was statistically significant only in the adjuvant setting (CALBG: Q5 vs. Q1: HR 2.32; 95% CI 1.36–3.96; *P* trend <0.001) [42], and not in the population-based study (NHS I: Q5 vs. Q1: HR 1.32 (0.89–1.97); *P* trend = 0.23) [23]. Similarly, a statistically significant increased risk of colon cancer recurrence was reported in the adjuvant setting [42], while a non-significant positive association was reported for CRC mortality in the population-based study [23] (Table 1). For the Prudent dietary pattern, both studies reported statistically non-significant associations for all-cause mortality [23, 42], CRC-specific mortality [23], or colon cancer recurrence [42].

Furthermore, several a priori-defined dietary patterns were studied in the two population-based studies [23, 24] (Table 1). Of the a priori-defined dietary patterns, none has been studied in more than one cohort. Some a priori-defined dietary patterns were associated with lower risk of all-cause mortality, but not all [23, 24].

Only one small (*n* = 111) randomized dietary intervention trial among CRC survivors assessed associations with survival [78]. Throughout the 1.5 months of neoadjuvant radiotherapy patients with rectal cancer randomized to the intervention group received 6 weekly individualized nutrition counseling and education sessions using regular foods, while the control group maintained their usual diet. Overall, the main goal of the intervention was to enable every patient to achieve his or her calculated energy and protein requirements. After long-term follow-up (median follow-up 6.5 (range 4.9–8.1) years), CRC-specific survival was significantly longer in the intervention group after adjustment for age and disease stage (median survival 7.3 vs. 4.9 years).

### Red and Processed Meats

Both NHS I [23] and CPS II Nutrition Cohort [40] reported on post-diagnosis red and processed meat intake, although the NHS I paper focused on dietary patterns (Table 1). The CPS II Nutrition Cohort also provided information regarding pre- to post-diagnosis change in red and processed meat consumption [40] (Table 2).

**Table 2:** Cohort studies among individuals with colon or rectal cancer examining change in lifestyle factors in relation to all-cause mortality, colorectal cancer-specific mortality, or recurrence; changes could be changes from pre- to post-diagnosis or changes made after diagnosis

First author, year, name of cohort, country	Study population	Time of post-diagnosis exposure assessment	Outcomes assessed	Year of CRC diagnosis and follow-up	Lifestyle factor	All-cause mortality HR (95% CI)	Colorectal cancer-specific mortality HR (95% CI)	Covariates
Change in diet—population-based studies								
McCullough, 2013, CPS II Nutrition Cohort, USA [40]	*n* = 1186 Both genders Colon and rectum Stage I–III	Red and processed meat 9 years before CRC diagnosis (mean) and 3 years after diagnosis (mean)	All-cause mortality (*n* = 472); CRC-specific mortality (*n* = 146); CVD-mortality (*n* = 110); other-mortality (*n* = 216)	Diagnosis 1992–2009; Mean 7.6 years (SD 3.4 years)	Remained low meat Remained high meat Low to high meat High to low meat	1.0 1.28 (0.98–1.67) 1.25 (0.93–1.67) 1.37 (1.02–1.85)	1.0 1.79 (1.11–2.89) 0.96 (0.55–1.66) 1.43 (0.80–2.57)	Age, sex, stage, 1992 pre-diagnostic energy intake, and post-diagnostic energy intake
Yang, 2017, CPS II Nutrition Cohort, USA [27]	*n* = 1599 M and W CRC Stage I–III	Alcohol 3.2 years before CRC diagnosis (mean) and 1.9 years after diagnosis (mean)	All-cause mortality (*n* = 732); CRC-specific mortality (*n* = 235); CVD-mortality (*n* = 172); other mortality (*n* = 325)	Diagnosis 1992–2011; Mean FU 8.2 years (SD 4.7 years)	Never drinker Former-former Current-former Current < 2 drinks/day Current ≥ 2 drink/day	1.0 1.09 (0.81–1.48) 1.21 (0.92–1.60) 0.94 (0.77–1.16) 0.92 (0.66–1.28)	1.0 1.28 (0.73–2.23) 1.81 (1.13–2.91) 1.27 (0.87–1.86) 1.44 (0.80–2.60)	Age, sex, tumor stage, smoking, BMI, PA, education, and pre-existing diseases in 1982/1992 (COPD, liver disease, kidney disease)
Change in physical activity—population-based studies								
Meyerhardt, 2006, Nurses’ Health Study I, USA [46]	*n* = 573 W only CRC Stage I–III	6 months before CRC diagnosis (mean) and 22 months after diagnosis (mean)	All-cause mortality (*n* = 132); CRC-specific mortality (*n* = 80)	Diagnosed 1986–2004; Median FU 9.6 years	Decreased PA No change in PA Increased PA MET-hours/week Stable activity < 9 Stable activity ≥ 9 Increase activity Pre-diagnosis activity < 9 Pre-diagnosis activity ≥ 9	1.23 (0.79–2.34) 1.0 0.51 (0.30–0.85) 1.0 0.33 (0.11–0.97) 0.26 (0.10–0.66) 0.35 (0.11–1.13)	1.32 (0.74–2.34) 1.0 0.48 (0.24–0.97) 1.0 0.27 (0.09–0.80) 0.36 (0.19–0.67) 0.62 (0.28–1.34)	BMI, stage, differentiation grade, tumor location, age, year of diagnosis, chemotherapy, time from diagnosis to PA measurement, change in BMI, smoking
Baade, 2011, Queensland, Australia [48]	*n* = 1825 M and W CRC Stage I–III	Pre-diagnosis physical activity was recalled after CRC diagnosis and assessed 5 months after diagnosis	All-cause mortality (*n* = 462); CRC-specific mortality (*n* = 345)	Diagnosis 2003–2004 Mean FU 4.9 years (range 4.0–6.0)	Pre-diagnosis to 5 months post-diagnosis No change or decreased Increased ≤ 2 h/week Increased > 2 h/week *P* trend	1.0 1.27 (0.88–1.83) 1.06 (0.65–1.71) 0.449	1.0 1.32 (0.89–1.98) 1.03 (0.59–1.80) 0.389	5 months post-diagnosis PA level, age, stage, smoking status, tumor location, treatment, sex and comorbidities
	*n* = 1554 M and W CRC Stage I–III	5 and 12 months after CRC diagnosis			5 to 12 months post-diagnosis No change or decreased Increased ≤ 2 h/wk. Increased > 2 h/wk. *P* trend	1.0 0.79 (0.59–1.04) 0.69 (0.50–0.94) 0.030	1.0 0.68 (0.48–0.97) 0.64 (0.44–0.93) 0.015	
Smoking cessation—population-based studies								
Phipps, 2011, Seattle Colon Cancer Family Registry, USA, [79]	*n* = 2264 M and W CRC Stage I–IV	2 years before CRC diagnosis and 8.0 months after diagnosis (mean)	All-cause mortality (*n* = 831); CRC-specific mortality (*n* = 562)	Diagnosis 1998–2007; FU up to 2010	Remained never smoker Remained former smoker Continued smoking Quit smoking	1.0 1.26 (1.07–1.48) 1.50 (1.14–1.97) 1.52 (1.21–1.90)	1.0 1.14 (0.93–1.38) 1.47 (1.07–2.03) 1.32 (1.00–1.74)	Age, time from diagnosis to interview, history of preventive screening, sex, and education level
Tao, 2013, Shanghai Cohort Study, China [28]	*n* = 114 M only CRC Stage NR	Yearly assessments of smoking cessation after diagnosis among smokers at diagnosis	All-cause mortality (*n* = 73)	Diagnosis 1986–2010; Mean FU 5.3 ± 4.8 years	Quit smoking Intermittent smoking Continued smoking Time-dependent smoking	1.0 1.35 (0.68–2.67) 3.46 (1.69–7.10) 2.31 (1.40–3.81)		Age, education, pack-years of smoking before diagnosis, treatment, and cancer site
Walter, 2015, DACHS study, Germany [58]	*n* = 3130 M and W CRC Stage I–IV	Smoking cessation in the year of diagnosis.	All-cause mortality (*n* = 889) CRC-specific mortality (*n* = 828), Non-CRC related mortality (*n* = 644), Recurrence-free survival (*n* = 1024), Disease-free survival (*n* = 232)	Diagnosis 2003–2010; Median FU 4.9 years.	Nonsmokers Recent quitters Continued smokers	1.0 0.97 (0.70–1.33) 1.10 (0.86–1.41)	1.0 0.87 (0.60–1.25) 1.10 (0.83–1.45)	Age, sex, BMI, stage, alcohol consumption, red meat consumption, family history of CRC, use of statins, use of NSAIDs, use of beta-blockers, diabetes mellitus, history of heart failure, myocardial infarction, angina pectoris or stroke, history of non-CRC cancer; additional adjustment for age × log(time) to account for time-dependent effects
Yang, 2015, CPS II Nutrition Cohort, USA [29]	*n* = 2256 M and W CRC Stage I–III	2.3 years before CRC diagnosis (mean) and 1.4 years after diagnosis (mean)	All-cause mortality (*n* = 859); CRC-specific mortality (*n* = 323)	Diagnosis 1992–2009; Mean FU 7.5 years (SD 4.6 years)	Remained never smoker Remained former smoker Continued smoking Quit smoking	1.0 1.18 (1.00–1.39) 2.33 (1.62–3.34) 1.94 (1.29–2.91)	1.0 0.86 (0.66–1.11) 2.20 (1.29–3.76) 1.85 (1.02–3.35)	Age, sex, stage, alcohol consumption, BMI, and PA
Weight change—population-based studies								
Baade, 2011, Queensland, Australia [48]	*n* = 1763 M and W CRC Stage I–III	Pre-diagnosis weight was recalled after CRC diagnosis and assessed 5 months after diagnosis	All-cause mortality (*n* = 462); CRC-specific mortality (*n* = 345)	Diagnosis 2003–2004 Mean FU 4.9 years (range 4.0–6.0)	Pre-diagnosis to 5 months post-diagnosis > 5 kg loss 2–4.9 kg loss ± 2 kg 2–4.9 kg gain > 5 kg gain *P* trend	1.63 (1.29–2.06) 1.10 (0.83–1.46) 1.0 1.12 (0.60–2.09) 1.63 (1.02–2.61) < 0.001	1.64 (1.24–2.15) 1.02 (0.73–1.42) 1.0 0.90 (0.41–1.96) 1.46 (0.84–2.53) 0.001	5 months post-diagnosis weight, height, PA level, stage, smoking status, tumor site, treatment, sex and comorbidities
	*n* = 1503 M and W CRC Stage I–III	5 months and 12 months after CRC diagnosis			5 to 12 months post-diagnosis > 5 kg loss 2–4.9 kg loss ± 2 kg 2–4.9 kg gain > 5 kg gain *P* trend	2.92 (1.89–4.49) 1.68 (1.10–2.59) 1.0 0.95 (0.68–1.32) 0.91 (0.69–1.20) < 0.001	3.21 (1.95–5.31) 1.59 (0.95–2.68) 1.0 1.02 (0.69–1.50) 0.89 (0.64–1.25) < 0.001	
Meyerhardt, 2016, Kaiser Permanente Northern Carolina, USA [80]	*n* = 2781 M and W CRC Stage I–III	Within 3 months after CRC diagnosis (prior to surgery) and approximately 18 months after diagnosis (range 15–21)	All-cause mortality (*n* = 549); CRC-specific mortality (*n* = 311)	Diagnosis 2006–2011; Median FU 4.2 years (range 0.1–8.1 years)	% Weight change ≥ 10% loss 5–9.9% loss ± 4.9% 5–9.9% gain ≥ 10% gain *P* trend loss *P* trend gain	3.27 (2.56–4.18) 1.74 (1.34–2.25) 1.0 0.86 (0.65–1.14) 1.20 (0.91–1.58) < 0.0001 0.27	3.20 (2.33–4.39) 1.58 (1.12–2.23) 1.0 0.84 (0.58–1.22) 0.93 (0.63–1.37) < 0.0001 0.54	Age, weight at diagnosis, gender, race/ethnicity, stage, grade, chemotherapy, and tumor site
Weight change—studies in the adjuvant setting								
Meyerhardt, 2008, CALGB 89803, USA [81]	*n* = 1053 M and W Colon Stage III	During and 6 months after adjuvant chemotherapy	All-cause mortality (*n* = 261); Recurrence-free survival (*n* = 338); Disease-free survival (*n* = 369)	Diagnosis 1999–2001; Median FU 5.3 years	Weight change (kg) > 5 loss 2.1–5 loss ± 2 2–4.9 gain ≥ 5 gain	1.13 (0.44–2.93) 0.89 (0.31–2.57) 1.0 0.97 (0.43–2.18) 1.23 (0.65–2.31)	† 1.35 (0.64–2.81) 1.04 (0.46–2.35) 1.0 1.00 (0.52–1.95) 1.17 (0.70–1.96)	Sex, age, T stage, number of positive lymph nodes, presence of clinical perforation at time of surgery, presence of bowel obstruction at time of surgery, performance status, treatment arm, time between questionnaire one and questionnaire two, time-varying BMI, smoking status at time of questionnaire two, PA level
Vergidis, 2016, British Columbia Cancer Agency, Canada [82]	*n* = 539 M and W Colon Stage III	At initial oncology consultation visit before the receipt of any systemic therapy and follow-up weights were serially reported at each subsequent clinic visit throughout the entire course of their adjuvant treatment or until 9 months after their first clinic visit, whichever came later. The peak and nadir weights were used to calculate weight change	All-cause mortality (*n* NR); Recurrence-free survival (*n* NR)	Diagnosis 2008–2010; FU 3–5 years	Weight gain < 5% ≥ 5% < 10% ≥ 10% Weight loss < 5% ≥ 5% < 10% ≥ 10% Weight change < 5% ≥ 5% < 10% ≥ 10%	1.0 0.80 (0.39–1.66) 1.0 0.52 (0.24–1.20) 1.0 1.92 (1.00–3.70) 1.0 2.63 (1.04–6.67) 1.0 1.02 (0.54–1.95) 1.0 1.15 (0.59–2.22)	† 1.0 0.84 (0.46–1.53) 1.0 0.81 (0.40–1.65) † 1.0 1.56 (0.88–2.28) 1.0 2.94 (1.39–6.25) † 1.0 1.44 (0.79–2.64) 1.0 1.10 (0.64–1.83)	Age, sex, comorbidities, performance status, tumor site, stage, grade, receipt of systemic therapy, type of regimen received
Muscle loss—studies in the metastatic setting								
Blauwhoff-Buskermolen, 2016, Vrije Universiteit Medical Center, the Netherlands [36]	*n* = 67 M and W CRC Stage IV	Skeletal muscle area was measured using CT scans conducted before start of chemotherapy and during chemotherapy	Overall mortality (*n* = NR)	Diagnosis 2011–2014; Median FU 17.5 months (95% CI 13.3–21.7) for patients receiving first-line chemotherapy and 8.5 months (95% CI 4.4–12.6) for patients receiving second-line chemotherapy or beyond	Muscle loss < 9% ≥ 9%	1.0 4.47 (2.21–9.05)		Sex, age, lactate dehydrogenase concentration, comorbidity, metastases, chemotherapy line, tumor progression at first evaluation by CT scan

*CRC* colorectal cancer, *HR* hazard ratio, *95% CI* 95% confidence interval, *M* men, *W* women, *NR* not reported, *BMI* body mass index, *PA* physical activity, *MET-h* metabolic equivalent task-hour, *CT* computed tomography, *CPS II* Cancer Prevention Study II, *CALGB* Cancer and Leukemia Group B, *DACHS* German: Darmkrebs: Chancen der Verhutüng durch Screening, English: chances for prevention through screening

^†^Results are for disease recurrence

These two studies did not observe an association between red and processed meat intake and both all-cause mortality and CRC-specific mortality [23, 40]. Furthermore, changing meat intake from high (median or higher) before CRC diagnosis to low (below median) after diagnosis was not associated with lower mortality when compared to survivors with a consistently high intake [40].

### Sugar-Sweetened Beverages

Both the NHS I [23] and CALGB 89803 [43] reported on post-diagnosis sugar-sweetened beverage intake and CRC outcomes (Table 1).

Both studies [23, 43] reported increased all-cause mortality risk for sugar-sweetened beverage consumption after CRC diagnosis, of which the association in the NHS I was statistically significant [23]. Each additional serving of sugar-sweetened beverages (including fruit juices) after CRC diagnosis was associated with an 11% increased risk for all-cause mortality (HR 1.11; 95% CI 1.01–1.23) [23]. A similar relative risk was reported for CRC-specific mortality, although it was not statistically significant [23]. For colon cancer recurrence, CALGB 89803 reported a statistically significant increased recurrence risk for patients consuming ≥ 2 servings of sugar-sweetened beverages per day (HR 1.75; 95% CI 1.04–2.94) compared to those consuming < 2 servings per month (*P* trend = 0.04) [43].

### Alcohol

Four population-based studies, NHS I [23, 44], HPFS [44], CPS II Nutrition cohort [27], and a Japanese cohort of CRC survivors [26], reported on post-diagnosis alcohol consumption and CRC outcomes (Table 1).

In NHS I, moderate drinking was used as the reference group and abstaining from alcohol consumption was associated with a statistically significant increased all-cause mortality risk (HR 1.30; 1.05–1.61) compared to women consuming 5–15 g of alcohol per day [23]. Drinking > 15 g/day (approximately 1.5 drinks) was not statistically significantly associated with increased mortality risk. Similarly, abstainers had a higher mortality risk than drinkers in the Japanese cohort [26] and after combining both NHS I and HPFS cohort data [44]. However, the CPS II Nutrition cohort reported that drinking alcohol after diagnosis was not associated with all-cause mortality [27]. For CRC-specific mortality, similar results were reported as for all-cause mortality (Table 1).

The CPS II Nutrition cohort also provided information regarding pre- to post-diagnosis change in alcohol consumption (Table 2). Participants who reported drinking before CRC diagnosis but stopped drinking alcohol after diagnosis had a statistically non-significant increased risk of all-cause and CRC-specific mortality compared to participants who continued to drink alcohol [27].

### Other Foods, Beverages, and Nutrients

The intake of some foods, beverages, and nutrients were only reported in one study each (Table 1). Higher nut consumption was associated with lower risk of CRC mortality (HR/serving/day 0.69; 95% CI 0.49–0.97) in the NHS I, while no statistically significant association was reported for all-cause mortality [23]. Furthermore, no associations were observed within the NHS I with either all-cause mortality or CRC-specific mortality for vegetables, fruits, or whole grains [23]. However, in the Japanese study, lower green leafy vegetable intake after CRC diagnosis was associated with an increased all-cause mortality risk [26].

Higher milk intake was statistically significantly associated with lower all-cause mortality risk (Q4 vs. Q1: HR 0.72; 95% CI 0.55–0.94; *P* trend = 0.02) in the CPS II Nutrition Cohort [41]. A similar risk was reported for overall dairy consumption, although associations did not reach statistical significance [41]. Additionally, higher coffee intake was statistically significantly associated with lower all-cause mortality (≥ 4 vs. 0 cups/day: HR 0.66; 95% CI 0.37–1.18; *P* trend = 0.01) within CALGB 89803 [25]. No significant associations were reported for non-herbal tea intake [25].

Higher dietary glycemic load and total carbohydrate intake were statistically significant associated with an increased risk of mortality and recurrence in CALGB 89803 [45]. Higher total calcium intake was statistically significantly associated with both lower all-cause mortality and CRC-specific mortality in the CPS II Nutrition Cohort, while no significant associations were reported for vitamin D [41]. Also no significant associations were reported for intake of one-carbon nutrients (folate, vitamins B_6_ and B_12_) in NHS I [44].

### Diet: Key Points

One small randomized intervention trial which provided individualized nutritional counseling and education about regular foods suggest that making dietary changes may improve cancer-specific survival. No dietary pattern or food has been studied in more than two observational cohorts, with cancer recurrence only studied in one cohort in the adjuvant setting embedded in a randomized chemotherapy trial. While alcohol consumption has been studied more frequently, these studies often used abstainers as comparison group. Abstainers are probably an inappropriate reference group, as this group may, at least in part, include people who stopped drinking because of comorbidities or cancer-related symptoms. Overall, emerging evidence shows that diet after CRC diagnosis might affect survival, but further research is needed to clarify what aspects of diet are important and which dietary changes could affect survival.

## Physical Activity after CRC Diagnosis

Seven population-based studies [26, 46–51] and one study in the adjuvant setting [52] provided results on physical activity after CRC diagnosis and mortality outcomes (Table 1). Five large US cohorts assessed post-diagnosis physical activity in population-based cohorts with > 500 CRC patients: NHS I [46], HPFS [47], CPS II Nutrition Cohort [50], Women’s Health Initiative [49], and National Institutes of Health-AARP Diet and Health Study [51]. All five cohorts consist of participants diagnosed with CRC during follow-up and have updated physical activity assessment after diagnosis, usually when treatment was completed. In contrast, two non-US cohorts (an Australian cohort [48] and BioBank Japan [26]) recruited > 1500 CRC patients after CRC diagnosis. All studies reported on leisure time physical activity.

### Physical Activity

For all-cause mortality, seven studies [26, 46–52] were included in previous meta-analyses [7–10]. These meta-analyses have found highest versus lowest post-diagnostic physical activity to be associated with 40% lower all-cause mortality risk [7–10]. Five studies that were included in a dose-response meta-analysis showed a 28% lower risk of all-cause mortality (HR 0.72; 95% CI 0.65–0.80) for every 10 metabolic equivalent task-hour per week (MET-hours/week) increase in post-diagnosis physical activity [9], which is equivalent to current recommendations of 150 min/week of at least moderate intensity activity. For CRC-specific mortality, similar risk reductions were reported comparing high versus low physical activity after CRC diagnosis (HR 0.62; 95% CI 0.45–0.86) [11••] and for every 10 MET-hours/week increase in post-diagnosis physical activity (HR 0.75; 95% CI 0.65–0.85) [9].

### Changes in Physical Activity

The Australian cohort [48] and NHS I [46] also provided results on changes in physical activity and mortality outcomes in CRC patients (Table 2). An increase of physical activity > 2 h/week between 5 and 12 months post-diagnosis was statistically significantly associated with lower all-cause (HR 0.69; 95% CI 0.50–0.94) and CRC-specific mortality (HR 0.64; 95% CI 0.44–0.93) among Australian CRC survivors [48]. A pre- to post-diagnosis increase in physical activity showed a statistically significant lower all-cause and CRC-specific mortality risk in the NHS I [46], but no association was reported among Australian CRC survivors [48] (Table 2). The first randomized controlled trial designed primarily to assess the impact of physical activity on survival among colon cancer survivors is ongoing [83]. As of April 2017, the trial has enrolled 536 of its planned 972 participants [84] and only 1 year feasibility results have been published so far [85].

### Sedentary Behavior

Three of the population-based studies, CPS II Nutrition Cohort [50], National Institutes of Health-AARP Diet and Health [51], and HPFS [53] also reported on post-diagnosis sedentary behavior and all-cause as well as CRC-specific mortality (Table 1). CPS II reported on leisure time spent sitting [50], whereas the other two studies assessed TV viewing [51, 53]. All three studies [50, 51, 53] reported no statistically significant associations between sedentary behavior and all-cause mortality. With regard to CRC-specific mortality, only one study, the CPS II Nutrition Cohort showed a statistically significant positive association between sedentary behavior and CRC-specific mortality (≥ 6 h vs. < 3 h/day sitting time: HR 1.62; 95% CI 1.07–2.44) [50].

### Physical Activity: Key Points

Evidence from prospective observational studies has consistently suggested that higher physical activity after CRC diagnosis is associated with a lower risk of CRC-specific and all-cause mortality, but whether physical activity is causally related to CRC mortality remains unclear. A randomized controlled trial is currently ongoing to address whether aerobic physical activity after complement of adjuvant therapy improves survival. Based on a few studies, there is some evidence suggesting that excessive sedentary behavior after CRC diagnosis might be associated with increased CRC-specific mortality, but findings are less consistent than for leisure time physical activity.

## Smoking after CRC Diagnosis

Eleven population-based studies [14, 26, 28–31, 54–58] and three studies in the adjuvant setting [59–61] reported on smoking at or after CRC diagnosis and mortality outcomes (Table 1). Four population-based studies used data from a cancer registry [14, 30, 31, 55]; three were from single-institution hospital cohorts [54, 56, 57]; three were non-US cohorts (Shanghai Cohort Study [28], the German cohort DACHS [58], and BioBank Japan [26]); and lastly, the CPS II Nutrition cohort [29]. Two studies in the adjuvant setting were embedded in an adjuvant chemotherapy trial, CALGB 89803 [60] and N0147 [61], while the third study included patients referred to a single institution for consideration of adjuvant treatment [59]. Six studies [28, 31, 54, 57–59] compared current smokers with non-smokers, while eight studies [14, 26, 29, 30, 55, 56, 60, 61] compared current smokers with never smokers.

### Smoking

For all-cause mortality, eight out of nine population-based studies [26, 28, 29, 31, 54–57] reported increased all-cause mortality risk for smoking, of which six [26, 28, 29, 54, 55, 57] were statistically significant. Furthermore, the study in the adjuvant setting also reported a statistically significant increased all-cause mortality risk for smoking [61].

For CRC-specific mortality, five population-based studies [14, 29, 30, 57, 58] reported increased CRC-specific mortality risk for smoking, of which three [14, 29, 30] were statistically significant (Table 1). However, one study that reported results separately for men and women reported a statistically non-significant positive association among women for post-diagnosis smoking, while among men, a statistically non-significant inverse association was reported [56]. Furthermore, one study in the adjuvant setting also reported a statistically significant increased CRC-specific mortality risk for smoking [59].

For colon cancer recurrence, one study embedded in the trial N0147 [61] reported a statistically significant increased cancer recurrence risk for smoking, while CALGB 89803 [60] reported no association with smoking among stage III colon cancer patients treated with adjuvant chemotherapy.

### Smoking Cessation

Four population-based studies provided results on smoking cessation and mortality outcomes in CRC patients (Table 2). People who continued smoking after CRC diagnosis had a more than threefold increased risk of all-cause mortality (HR 3.46; 95% CI 1.69–7.10) compared to people who quit smoking after diagnosis [28]. Pre- to post-diagnosis smoking cessation was not statistically significantly associated with all-cause or CRC-specific mortality risk [29, 58, 79], although one of these studies reported lower mortality risk for those who quit smoking compared to those who continued to smoke [29].

### Smoking: Key Points

Overall, evidence from observational studies has consistently suggested that smoking after CRC diagnosis increases the risk of CRC-specific and all-cause mortality. It seems plausible that smoking cessation would improve survival outcomes in CRC survivors, although direct evidence is limited.

## Body Fatness and Body Composition after CRC Diagnosis

This review first focusses on studies that assessed BMI at or after CRC diagnosis. Next, we discuss weight changes and lastly, we describe the results of studies which quantified visceral adipose tissue or skeletal muscle mass from CT images.

### Body Mass Index

Eleven population-based studies [16, 26, 32, 33, 48, 49, 62–66], two studies from adjuvant chemotherapy trials [67, 68], and one study among metastatic patients [34] assessed the association of BMI at or after CRC diagnosis and CRC outcomes (Table 1). Furthermore, 21 additional studies in the adjuvant setting were included in a pooled analyses of patients enrolled in trials of adjuvant chemotherapy [69]. Moreover, an additional article with pooled analyses in the metastatic setting included data of 25 treatment trials [70].

For underweight (either BMI < 18.5 or 20 kg/m^2^), all population-based studies [16, 26, 32, 33, 48, 62–65], the pooled analysis of studies in the adjuvant setting [69], and both publications in the metastatic setting [34, 70] reported higher all-cause mortality risk compared to normal weight individuals. The majority of these studies [26, 32, 34, 48, 62, 65, 69, 70] reported statistically significant results (Table 1). In the largest population-based study, ~ 3400 men and women diagnosed with stage I to III CRC from the Kaiser Permanente Northern California population, underweight at diagnosis was associated with a threefold increased all-cause mortality risk (HR 3.01; 95% CI 1.88–4.83) compared to normal weight [32]. However, most other studies report a 1.5- to 2-fold increased risk (Table 1). Generally, similar results were reported for CRC-specific mortality and cancer recurrence (Table 1).

For overweight (defined as BMI 25.0–24.9 kg/m^2^), all population-based studies [16, 26, 32, 33, 48, 49, 62, 64–66] reported lower all-cause mortality risk compared to normal weight individuals, of which three were statistically significant [48, 49, 62]. However, studies in the adjuvant setting of a chemotherapy trial reported that overweight individuals had a similar all-cause mortality risk as normal weight individuals (Table 1). For metastatic patients participating in treatment trials, all-cause mortality risk was lowest at BMI 28 kg/m^2^ [70], while overweight was associated with an increased all-cause mortality risk among a general population of patients diagnosed with metastatic disease (HR 1.23; 95% CI 1.03–1.46) [34]. Generally, similar results were reported for CRC-specific mortality and cancer recurrence (Table 1).

For obesity (BMI ≥ 30 kg/m^2^), none of the population-based studies [16, 26, 32, 33, 48, 49, 62, 64–66] reported statistically significant associations with all-cause mortality. Nevertheless, the only study (Kaiser Permanente Northern California cohort) that reported on a separate group with class II or III obesity (BMI ≥ 35 kg/m^2^) reported a statistically significant increased all-cause mortality risk [32]. Within the adjuvant setting pooled analyses showed a modest increased all-cause mortality risk (HR 1.10; 95% CI 1.04–1.17) compared with normal weight [69]. Within the metastatic setting, both publications showed that obese individuals had a somewhat similar, or lower, all-cause mortality risk as normal weight individuals [34, 70]. Generally, similar results were reported for CRC-specific mortality and cancer recurrence (Table 1).

### Changes in Weight

Four studies [48, 80–82] reported on weight changes (Table 2). Two studies were population-based studies, a cohort from the Kaiser Permanente Northern California population [80] and an Australian cohort [48], and two studies were in the adjuvant setting, CALGB 89803 [81] and a cohort from the British Columbia Cancer Agency [82].

Large post-diagnosis weight loss (> 5 kg or ≥ 10%) was associated with a threefold increased all-cause and CRC mortality risk compared with stable weight in both population-based studies [48, 80]. Modest weight loss (2–4.9 kg or 5–9.9%) was also associated with increased all-cause and CRC mortality risk [48, 80], although only statistically significant in the Kaiser Permanente Northern California cohort [80]. In fact, the association between weight loss and mortality was present regardless of at-diagnosis BMI [80]. Large weight loss during adjuvant chemotherapy was associated with increased all-cause mortality and recurrence risk in a cohort from the British Columbia Cancer Agency [82], but not in CALGB 89803 [81].

Post-diagnosis weight gain was not associated with increased all-cause or CRC-specific mortality risk [48, 80, 81] or colon cancer recurrence [81, 82]. Furthermore, pre- to post-diagnosis weight loss or weight gain of >5 kg were both associated with a statistically significant 60% higher all-cause risk compared to stable weight [48].

### Visceral Adipose Tissue

Three population-based studies [35, 39, 71], two studies in the adjuvant setting [72, 73], and one study among metastatic patients [74] reported on post-diagnosis visceral adipose tissue and all-cause mortality (Table 1). Most of these studies were small (*n* = 62 to 339), except for the population-based cohort from the Kaiser Permanente Northern California population (*n* ~ 3200) [39••].

For all-cause mortality, all population-based studies [35, 39••, 71] reported statistically non-significant associations with visceral adipose tissue (Table 1). Both studies among patients treated with chemotherapy [72, 73] reported an increased all-cause mortality risk with high visceral adipose tissue, of which one was statistically significant [72]. The study among metastatic CRC patients [74] reported a statistically significant increased all-cause mortality risk for high visceral adipose tissue among patients treated with chemotherapy plus the angiogenesis inhibitor bevacizumab, but not among patients treated with chemotherapy only.

### Skeletal Muscle Mass

Four population-based studies [35, 38, 39••, 75], one study in the adjuvant setting [37] and three studies among patients with metastatic disease [36, 76, 77] reported on all-cause mortality (Table 1). Most of these studies were small (*n* = 67 to 339), except two population-based cohorts, from the Kaiser Permanente Northern California population (*n* ~ 3200) [39••] and from a single-institution hospital cohort that included stage I–IV patients [38].

Seven out of eight studies [36–38, 39••, 75–77] reported increased all-cause mortality risk for low skeletal muscle mass, of which five were statistically significant [37, 38, 39••, 75, 76] (Table 1). A meta-analysis, based on three small studies [75–77], concluded that a low muscle mass was statistically significantly associated with a more than twofold increased all-cause mortality risk (HR 2.25; 95% CI 1.63–3.09) [20]. The only large population-based cohort with non-metastatic patients, from Kaiser Permanente Northern California, showed an almost 30% increased risk of overall mortality and 50% increased risk of CRC-specific mortality [39••].

One study among metastatic patients reported on loss of muscle mass during chemotherapy [36]. This study showed that ≥ 9% loss of muscle mass during chemotherapy was associated with a more than fourfold increased all-cause mortality risk (HR 4.47; 95% CI 2.21–9.05) [36].

### Body Fatness and Body Composition: Key Points

Body fatness was studied most often by assessment of body mass index, while only few studies assessed other measures of body composition. Altogether, the results of studies across the three study categories (population-based, adjuvant, and metastatic setting) suggest a J- or L-shaped association between BMI and all-cause mortality or CRC-specific mortality risk. The risk of death was highest among patients who were underweight, while lowest risk was seen in patients with a BMI between 25 and < 30 kg/m^2^. If obesity confers an additional mortality risk compared to normal weight or overweight patients remains uncertain. Nevertheless, the most recent meta-analysis of post-diagnosis BMI concluded that obesity was statistically significantly associated with a modest 8% increased all-cause mortality risk (HR 1.08; 95% CI 1.03–1.13) compared to normal weight, while no association was found between obesity and CRC-specific mortality [17••]. Weight loss in the first 2 years after diagnosis was consistently associated with increased mortality risk and this association was independent of BMI at CRC diagnosis. Currently, there are no intentional weight loss trials among CRC survivors that assessed mortality risk [86••] and no study that assessed the effect of weight loss after treatment was successfully completed. That being overweight, and in some studies even obese states, seem to be associated with improved survival compared to normal weight is called the “obesity paradox.” The obesity paradox could be explained by several methodological issues, including the crudeness of BMI as a measure of body fatness, especially in a cancer patient population where loss of weight and lean body mass is a strong adverse factor [87].

Other measures used to study the association between body composition and CRC outcomes were visceral adipose tissue and muscle mass quantified from CT images; studies with other measures, such as waist circumference, are currently lacking. There is only limited evidence that visceral adiposity increased mortality risk. Across study categories, studies had mixed results. Only in the adjuvant setting, two small studies consistently showed increased all-cause mortality risk with higher visceral adipose tissue. Even though quantification of adipose tissue from CT scans is regarded as a more precise measure of adiposity than BMI, the usefulness of single-slice analysis might be limited [88]. On the other hand, evidence consistently shows that low muscle mass is associated with reduced survival, although each study used other cut points to define low muscle mass. The notion that the association between overweight and lower mortality is due solely to methodologic biases is refuted by results from the only large population-based study among non-metastatic CRC patients with available data for both BMI and body composition [39••]. Within the overweight BMI range between 25 and < 30 kg/m^2^, body composition appeared to explain why a BMI higher than normal is associated with the lowest mortality. The majority (78%) of patients in the overweight group had adequate muscle mass, while less than half (43%) of the patients with a normal BMI had adequate muscle mass. Furthermore, the obesity paradox could also be explained by clinical issues [87], such as metabolic health. One study at Kaiser Permanente investigated the combination of obesity and metabolic health and concluded that mortality risk was statistically significantly increased in obese patients with the metabolic syndrome, but not in metabolically healthy obese patients, compared with metabolically healthy non-obese patients [89].

## Conclusions and Future Directions

In conclusion, this review suggests that some, albeit not all, modifiable risk factors for cancer incidence might also be associated with mortality risk after CRC diagnosis. CRC prognosis appears to be worse with increased physical inactivity, smoking, or being underweight after CRC diagnosis. Emerging evidence suggests that diets associated with a positive energy balance, e.g., high consumption of sugar-sweetened beverages, may negatively impact survival in CRC survivors. Nonetheless, data relating post-diagnosis diet to CRC prognosis are scarce; with less than three observational studies that have examined associations for each dietary pattern or individual food after CRC diagnosis. In contrast, high red and processed meat or alcohol intake, established risk factors for incident CRC, do not appear to be associated with mortality after CRC diagnosis. Whether overweight and obesity after CRC diagnosis might confer an additional mortality risk compared to normal weight is still controversial and might depend on how body fatness is assessed and whether muscle mass was accounted for.

Since the first review on lifestyle factors in CRC survivors in 2010 [90], many new studies in this evolving area of research were published and summarized in subsequent reviews and meta-analyses. This is the first paper to comprehensively review post-diagnosis diet, physical activity, smoking, and body composition together in one review. Our findings were generally consistent with previous work, regarding diet [4••], physical activity [7–10, 11••], smoking [13••], and underweight [16, 17••, 19], although we included new publications. Overweight, assessed by BMI, was consistently associated with lowest mortality risk, although discussion remains about the causal claims regarding the effects of BMI on post-diagnosis mortality for CRC survivors. The only large population-based study among non-metastatic CRC patients concluded that body composition, i.e., muscle mass, appeared to explain why a BMI higher than normal is associated with the lowest mortality risk [39••]. Moreover, low muscle mass was consistently associated with increased mortality risk. Besides observational data, there were no reported randomized controlled trials in smoking or alcohol cessation/reduction, while physical activity and/or dietary/excess weight interventions only reported on short-term outcomes [86••]. Only one small randomized trial assessed long-term follow-up among CRC survivors, finding significantly improved cancer-specific survival after dietary counseling [78].

As people do not have isolated behaviors, a multidimensional lifestyle approach would be most informative for exploring mortality risk and cancer recurrence, as well as for translating these findings into meaningful strategies to improve disease prognosis. Some randomized controlled trials with both dietary and physical activity components have included CRC survivors, but they usually did not test the impact of comprehensive lifestyle interventions on risk of cancer recurrence or survival [86••]. Furthermore, only one observational study evaluated the association of post-diagnosis comprehensive lifestyle patterns and CRC outcomes [91]. That study concluded that adherence to the WCRF recommendations on diet, physical activity, and body fatness was not statistically significantly associated with mortality [91]. However, lifestyle was assessed on average 9 years after diagnosis and survivors were therefore at low risk to die from CRC during subsequent follow-up. Further research on post-diagnosis lifestyle patterns is needed to understand the multifactorial nature of risk of mortality and cancer recurrence and, furthermore, to avoid overemphasis of single lifestyle factors.

The existing studies have several limitations. Few observational studies have reported on the association between post-diagnostic lifestyle and CRC outcomes adjusting for pre-diagnostic lifestyle; thus, it is unknown whether the observed associations between post-diagnostic lifestyle and survival are independent of pre-diagnosis lifestyle. Furthermore, only few studies assessed changes in lifestyle over time in relation to CRC outcomes, with weight change and smoking cessation studied most often. Large prospective cohort studies, such as NHS I, HPFS, the COLON study [92], and others [93, 94], provide further opportunities to examine post-diagnosis lifestyle changes in relation to CRC prognosis during different phases of the cancer trajectory.

Studies evaluating lifestyle factors and CRC outcomes mainly focused on mortality, while cancer recurrence and comorbidities are other important outcomes. Disease recurrence was usually reported by studies in the adjuvant setting, but is not commonly reported by population-based studies. Furthermore, definitions of recurrence were inconsistent between studies. Using the standard definitions proposed by Punt et al. [95] may add to the cross-comparability of future studies. In addition, few studies among CRC survivors studied incidence and progression of comorbidities, although some studies included cardiovascular-mortality as an endpoint. Only one study assessed the incidence of comorbidities after CRC diagnosis [96]. This study observed that BMI and sedentary behavior at 5 months post-diagnosis were associated with the development of comorbid cardiovascular disease in the first 3 years after CRC diagnosis.

More research is needed on the mechanisms underlying the impact of lifestyle after CRC diagnosis on prognosis. A lifestyle contributing to a positive energy balance and hyperinsulinemia has been suggested to be implicated in the prognosis of CRC [5, 97]. For instance, determinants of hyperinsulinemia, such as physical inactivity, excessive sedentary behavior, and several aspects of diet, are associated with increased mortality risk. The dietary factors included in this review that might be linked to insulin-related pathways, a Western dietary pattern [23, 42], sugar-sweetened beverages [23, 43], low coffee consumption [25], and higher dietary glycemic load [45] all showed increased mortality risk. Also, a high-insulinogenic diet [98] has been associated with increased mortality risk. However, these studies were almost all conducted in the same cohort embedded in a trial of adjuvant chemotherapy (CALGB 89803) [25, 42, 43, 45].

Overall, evidence is emerging that modifiable lifestyle factors after CRC diagnosis, such as physical activity, smoking, body composition, and diet could impact survival. Although, not all modifiable risk factors for cancer presentation seem relevant for cancer survivors. With increasing CRC survivorship, however, CRC recurrence should be studied as a key outcome within population-based studies of CRC survivors. Additionally, studies are needed to evaluate the development and progression of comorbidities after CRC diagnosis. Studying lifestyle patterns over time, by including multiple lifestyle factors simultaneously at different time points during the cancer trajectory, would lead to a greater understanding of the multifactorial influence on CRC prognosis. Additional data from prospective observational studies and randomized controlled trials are urgently needed and, ultimately, will allow for lifestyle recommendations that are specifically tailored to cancer survivors.
